# Pulmonary Hypertension: Molecular Mechanisms and Clinical Studies

**DOI:** 10.1002/mco2.70134

**Published:** 2025-03-10

**Authors:** Joseph Adu‐Amankwaah, Qiang You, Xiaoer Liu, Jiayi Jiang, Dongqi Yang, Kuntao Liu, Jinxiang Yuan, Yanfang Wang, Qinghua Hu, Rubin Tan

**Affiliations:** ^1^ Department of Physiology Basic Medical School Xuzhou Medical University Xuzhou China; ^2^ School of Pharmacy Shandong University of Traditional Chinese Medicine Jinan Shandong China; ^3^ The First Clinical Medicine College Xuzhou Medical University Xuzhou China; ^4^ College of Life Science Xuzhou Medical University Xuzhou China; ^5^ Lin He's Academician Workstation of New Medicine and Clinical Translation Jining Medical University Jining China; ^6^ State Key Laboratory of Animal Biotech Breeding Institute of Animal Science Chinese Academy of Agricultural Sciences Beijing China; ^7^ Department of Pathophysiology School of Basic Medicine Tongji Medical College Huazhong University of Science and Technology Wuhan China; ^8^ Key Laboratory of Pulmonary Diseases of Ministry of Health Tongji Medical College Huazhong University of Science and Technology Wuhan China

**Keywords:** clinical therapeutics, epigenetic, genetic, noncoding RNAs, pulmonary hypertension

## Abstract

Pulmonary hypertension (PH) stands as a tumor paradigm cardiovascular disease marked by hyperproliferation of cells and vascular remodeling, culminating in heart failure. Complex genetic and epigenetic mechanisms collectively contribute to the disruption of pulmonary vascular homeostasis. In recent years, advancements in research technology have identified numerous gene deletions and mutations, in addition to *bone morphogenetic protein receptor type 2*, that are closely associated with the vascular remodeling process in PH. Additionally, epigenetic modifications such as RNA methylation, DNA methylation, histone modification, and noncoding RNAs have been shown to precisely regulate PH molecular networks in a cell‐type‐specific manner, emerging as potential biomarkers and therapeutic targets. This review summarizes and analyzes the roles and molecular mechanisms of currently identified genes and epigenetic factors in PH, emphasizing the pivotal role of long ncRNAs in its regulation. Additionally, it examines current clinical and preclinical therapies for PH targeting these genes and epigenetic factors and explores potential new treatment strategies.

## Graphical abstract

The figure highlights the interplay of genetic and epigenetic mechanisms in pulmonary hypertension (PH) and their implications for therapeutic strategies. The genetic mechanism section features key genes implicated in PH pathogenesis. The epigenetic mechanism encompasses DNA methylation, RNA methylation, noncoding RNAs (miRNA, lncRNA), and histone modifications. These insights inform targeted therapeutic strategies to address PH.

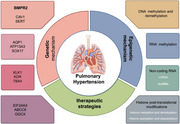

## 1 Introduction

Pulmonary hypertension (PH) refers to a clinical and pathophysiological syndrome characterized by changes in pulmonary vascular structure or function caused by various heterogeneous diseases (etiologies) and different pathogenesis, leading to elevated pulmonary vascular resistance (PVR) and mean pulmonary arterial pressure (mPAP), which can then develop into right heart failure or even death [1, 2, 3]. In 2018, approximately 25,000 deaths were attributed to PH [4]. This escalation in PH poses notable economic and social impacts, straining health systems globally [5]. Socially, it underscores the need for awareness, early diagnosis, and effective management to alleviate the burden on individuals and communities.

The latest diagnostic criteria for PH are primarily based on the measurement of mPAP and PVR, as well as pulmonary artery wedge pressure (PAWP) in specific subgroups. PH is defined by an mPAP greater than 20 mmHg. For precapillary PH, the mPAP exceeds 20 mmHg, with a PAWP of 15 mmHg or lower, and a PVR greater than 2 Wood units (WU). In contrast, the diagnosis of isolated postcapillary PH involves an mPAP greater than 20 mmHg, with a PAWP greater than 15 mmHg and a PVR of 2 WU or lower. Combined pre‐ and postcapillary PH is characterized by an mPAP exceeding 20 mmHg, a PAWP greater than 15 mmHg, and a PVR greater than 2 WU. Additionally, exercise‐induced PH is identified by an mPAP/CO slope greater than 3 mmHg/L/min during physical exertion. These hemodynamic parameters are essential for accurately diagnosing PH and determining its specific etiology [6].

**FIGURE 1:**
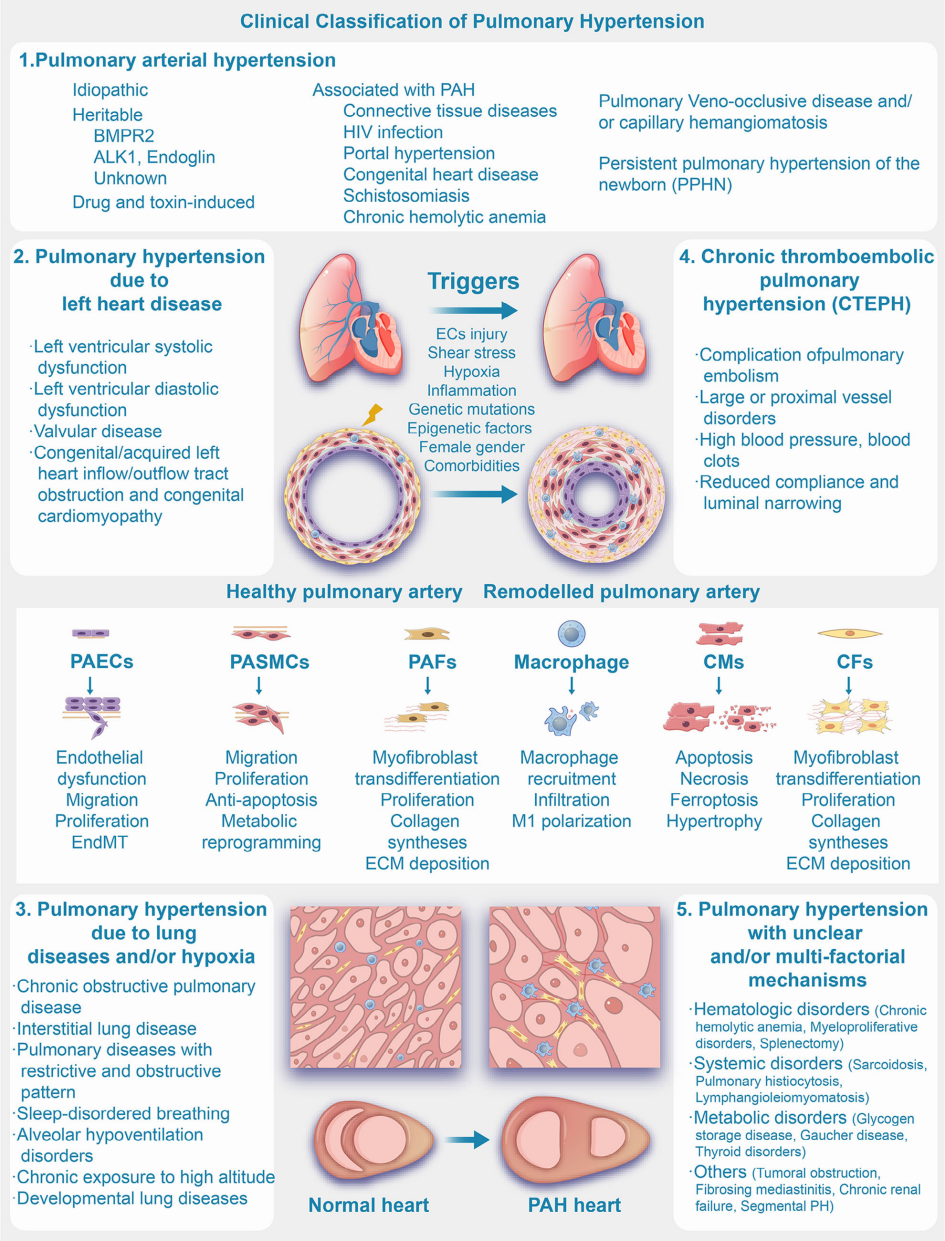
WHO group classification and the pathologic characteristics of PH. PAH, pulmonary arterial hypertension; PAECs, pulmonary artery endothelial cells; PASMCs, pulmonary artery smooth muscle cells; PAFs, pulmonary arterial fibroblasts; HIV, human immunodeficiency virus; CMs, cardiomyocytes; CFs, cardiac fibroblasts.

Based on the revised clinical classification as outlined in the 2022 ESC/ERS Guidelines, PH is classified according to underlying causes, pathophysiological mechanisms, clinical presentation, and therapeutic management. This updated classification provides a comprehensive framework to better understand the diverse etiologies of PH, guide clinical decision‐making, and optimize treatment strategies [6]. Group 1 is pulmonary arterial hypertension (PAH), which encompasses idiopathic, heritable, and drug‐induced forms, along with other associated conditions such as connective tissue disease, human immunodeficiency virus (HIV) infection, and congenital heart disease (CHD). Group 3 has evolved, with the term “sleep‐disordered breathing” replaced by “hypoventilation syndromes” to better capture conditions that cause daytime hypercapnia and are associated with an increased risk of PH. Group 4 includes PH associated with pulmonary artery obstructions, such as chronic thromboembolic pulmonary hypertension (CTEPH) and other forms of pulmonary artery obstruction. Last, Group 5 encompasses PH with unclear and multifactorial mechanisms, including conditions like hematological disorders, metabolic disorders, chronic renal failure, and fibrosing mediastinitis. This classification highlights the complexity of PH and its diverse etiologies, guiding both diagnosis and management (Figure 1) [6, 7].

Elevations in PVR and PAP are caused by pulmonary vascular remodeling and sustained pulmonary vasoconstriction in patients with PH [8, 9]. The process of pulmonary artery remodeling in PH involves several key events. These include the endothelial‐to‐mesenchymal transition (EndMT) and excessive proliferation of injured pulmonary artery endothelial cells (PAECs) in the microvasculature, as well as the overproliferation and phenotypic transformation of pulmonary artery smooth muscle cells (PASMCs). Furthermore, fibroblast activation results in the deposition of extracellular matrix (ECM), and the concurrent loss of capillaries, along with increased inflammatory cell infiltration around blood vessels and calcium dysregulation, contributes to the remodeling process. These collective alterations lead to vascular wall thickening, vessel lumen narrowing, elevated PVR, and the overall advancement of PH (Figure 1) [7, 8, 9, 10, 11, 12, 13]. Ultimately, this progression may culminate in right ventricular hypertrophy (RVH), fibrosis (Figure 1), heart failure in severe instances, and mortality [2, 14, 15].

The pathogenesis of PH involves intricate genetic and epigenetic mechanisms that contribute to the dysregulation of pulmonary vascular homeostasis. Genetic alterations, including mutations and polymorphisms in genes associated with vascular remodeling and smooth muscle function, have been implicated in the development of PAH. Epigenetic mechanisms, such as DNA methylation and histone posttranslational modifications (PTMs), play critical roles in regulating gene expression without altering the underlying DNA sequence [16, 17, 18]. Aberrant DNA methylation patterns can lead to the silencing of tumor suppressor genes and the activation of oncogenes, promoting cellular proliferation and migration in pulmonary vascular cells. Furthermore, histone modifications, including acetylation and methylation, influence chromatin structure and accessibility, thereby modulating the transcriptional landscape of genes involved in vascular tone regulation and inflammation. Additionally, noncoding RNAs (ncRNA), including microRNAs (miRNAs) and long ncRNAs (lncRNAs), significantly influence gene expression by targeting mRNAs for degradation or translational repression [17, 18]. In the last decade, studies have found that lncRNA plays a role in regulating gene expression, including gene silencing, chromatin modification, epigenetic gene expression, transcription or PTM, and translation [19], thus participating in pathophysiological processes of PH such as cell proliferation, cell cycle regulation, and apoptosis [20]. These epigenetic and genetic alterations collectively contribute to the remodeling of pulmonary vasculature, endothelial dysfunction, and the progression of PAH.

Therefore, in this comprehensive review, we explored the genetic and epigenetic mechanisms contributing to the pathogenesis of PH, with a particular focus on lncRNAs, and discussed their potential role in the clinical management and progression of PH. Specifically, we provided an in‐depth analysis of 27 verified lncRNAs in PH, drawing evidence from the literature to shed light on their targets and mechanisms. Moreover, RNA therapy during the coronavirus disease 2019 (COVID‐19) pandemic has marked a significant development in the pharmaceutical industry, acknowledged with the Nobel Prize in Physiology or Medicine. lncRNAs have emerged as one of the most promising targets in this field. Consequently, we proposed therapeutic strategies targeting lncRNAs for PH and highlighted their potential as biomarkers for diagnosis and prognosis. Last, we provided an update on the clinical journey of PH, discussing emerging therapies, novel targets in PH management, and the efficacy and safety of approved pharmacological therapies.

## 2 Genetic Mechanisms in the Pathogenesis of PH

### 2.1 Bone Morphogenetic Protein Receptor Type 2

**FIGURE 2:**
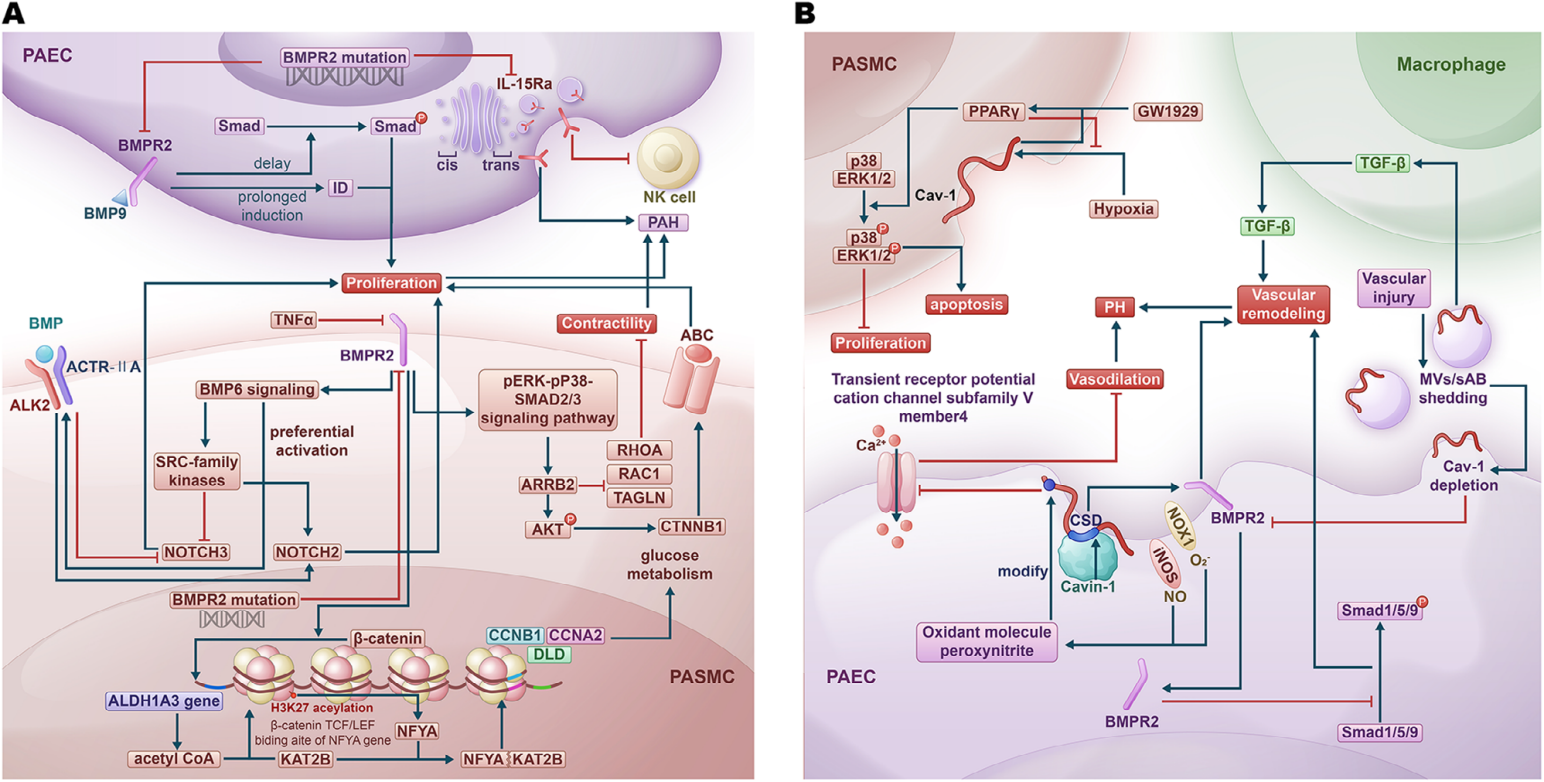
The role of *BMPR2* and *CAV‐1* in PAH. (A) The role of *BMPR2* in PH. In PAECs and PASMCs, mutations in *BMPR2* lead to a reduction in BMPR2 protein levels, disrupting the BMP signaling pathway and promoting excessive cell proliferation. Additionally, in PAECs, *BMPR2* mutations contribute to PH by inhibiting IL‐15Ra expression at the cell membrane through disruption of the trans‐Golgi network. Furthermore, in PASMCs, reduced BMPR2 levels affect histone methylation, leading to increased NFYA expression, which in turn enhances cell cycle progression and glycolytic protein expression, further driving pathological remodeling in PH. (B) The role of *CAV‐1* in PH. Similarly, a decrease in Cav‐1 at the cell membrane in both PAECs and PASMCs contributes to the development of PH. In PAECs, the oxidant molecule peroxynitrite, produced by NOX1 and iNOS, modifies Cav‐1, leading to reduced calcium influx in vascular endothelial cells. This inhibits vasodilation and promotes PH. Additionally, Cavin‐1 competitively binds to Cav‐1, reducing BMPR2 levels at the cell membrane, thereby inhibiting its downstream signaling and driving vascular remodeling. Vascular injury‐induced shedding of MVs and sAB depletes Cav‐1, further decreasing BMPR2 levels. This shedding also enhances TGF‐β production by macrophages, contributing to disease progression. In PASMCs, activation of PPARγ by the agonist GW1929 requires Cav‐1 and leads to increased p38 and ERK1/2 phosphorylation, promoting apoptosis while inhibiting proliferation. Moreover, PPARγ activation by GW1929 significantly reduces hypoxia‐induced Cav‐1 upregulation, establishing a negative feedback mechanism that regulates its expression. PAEC, pulmonary artery endothelial cell; PASMC, pulmonary artery smooth muscle cell; PAH, pulmonary arterial hypertension; PH, pulmonary hypertension; PAEC, pulmonary artery endothelial cell; PASMC, pulmonary artery smooth muscle cell; PAH, pulmonary arterial hypertension; PH, pulmonary hypertension; GW1929, a PPARγ agonist; MVs, microvesicles; sAB, small apoptotic bodies; CSD, caveolin scaffolding domain; NK, cell natural killer cell.

*Bone morphogenetic protein receptor type 2* (*BMPR2*) gene plays an important role in PAH (Figure 2A) [21, 22]. Loss of BMPR2 enhances the proliferation response of endothelial cells to bone morphogenetic protein (BMP) 9 by delaying the phosphorylation of Smad proteins and extending the induction of inhibitor of DNA binding [23]. The reduction of BMPR2 mediated by tumor necrosis factor‐α (TNF‐α) induced BMP6 signaling, which preferentially activates ALK2 (activin receptor‐like kinase 2)/ACTR‐IIA (activin a receptor type 2A) signaling axis and upregulates SRC proto‐oncogene, nonreceptor tyrosine kinase‐family kinases. Both pathways induce notch receptor (NOTCH) 2 while inhibiting NOTCH3, thereby promoting the proliferation of PASMCs in heritable pulmonary arterial hypertension (HPAH) [24]. BMPR2 deficiency resulting from a *BMPR2* mutation leads to the upregulation of the phosphorylated extracellular signal‐regulated kinase–phosphorylated mitogen‐activated protein kinase (MAPK)–SMAD2/3 signaling pathway. This, in turn, increases the levels of arrestin beta 2 (ARRB2). As a consequence, ARRB2 elevates catenin beta (CTNNB1) by enhancing phosphorylated AKT serine/threonine kinase 1 activity, which promotes PASMC proliferation via ATP‐binding cassette (ABC) activation. Simultaneously, this process reduces the contractility of PASMCs by downregulating ras homolog family member A (RhoA), Rac family small GTPase 1, and transgelin [25]. Additionally, the *BMPR2* mutation reduces BMPR2 levels and decreases IL (interleukin)‐15Rα surface presentation by affecting the trans‐Golgi network, which reduces natural killer (NK) cell numbers and exacerbates PH [26]. In PAH‐PASMCs with BMPR2 loss, β‐catenin drives an increase in *aldehyde dehydrogenase 1 family member A3* gene expression, resulting in elevated levels of acetyl coenzyme A (acetyl CoA) in the nucleus. This rise in acetyl CoA, in conjunction with lysine acetyltransferase 2B (KAT2B), leads to the acetylation of lysine 27 on histone 3 (H3K27) at the β‐catenin/TCF (T‐cell factor)/LEF1 (lymphoid enhancer‐binding factor 1) binding site of the *nuclear transcription factor Y subunit α* (*NFYA*) gene, thereby increasing the expression of NFYA. As a result, NFYA binds to KAT2B and stimulates cellular proliferation and glucose metabolism by promoting the expression of genes associated with the cell cycle and glucose metabolism, including *cyclin B1*, *cyclin A2* (*CCNA2*), and *dihydrolipoamide dehydrogenase* [27].

### 2.2 Caveolin‐1

Caveolin‐1 (Cav‐1), encoded by *CAV1*, is a component of the caveolae in the cell membrane [28], which has been shown to be associated with many lung diseases, including PH (Figure 2B) [29]. Inducible nitric oxide synthase and NADPH oxidase 1 (NOX1) colocalize with Cav‐1 to produce the oxidant molecule peroxynitrite, which inhibits the transient receptor potential cation channel subfamily V member 4 through cysteine modifications on Cav‐1. This inhibition reduces calcium influx mediated by this channel, which diminishes vasodilation and ultimately contributes to PH [30]. The peroxisome proliferator‐activated receptor (PPAR) γ agonist GW1929 activated PPARγ in a Cav‐1‐dependent manner, leading to increased phosphorylation of p38 and ERK1/2, which promoted apoptosis and inhibited the proliferation of PASMCs. Additionally, GW1929 significantly reduced the hypoxia‐induced upregulation of Cav‐1, demonstrating a negative feedback mechanism in this pathway [31]. Additionally, there are associations between Cav‐1 and BMPR2 in the pathogenesis of PH. Vascular injury triggers the shedding of microvesicles and small apoptotic bodies from epithelial cells, which enhances transforming growth factor‐β (TGF‐β) production by macrophages and subsequently upregulates pSMAD2/3. This shedding also leads to the depletion of Cav‐1, resulting in reduced expression of BMPR2, ultimately contributing to vascular remodeling and the development of PAH [32]. Caveolae associated protein 1 (Cavin‐1) competes with BMPR2 for binding to the caveolin scaffolding domain of Cav‐1 at the cell membrane. Consequently, increased levels of Cavin‐1 or the knockdown of *Cav‐1* facilitate the translocation of BMPR2 from the membrane to the cytoplasm. This translocation inhibits the phosphorylation of Smad1/5/9, promoting pulmonary vascular remodeling and ultimately contributing to the development of PH [33]. In the lungs of mice infected with *Schistosoma mansoni*, hyperphosphorylation of Cav‐1, along with reduced expression of Cav‐1 and BMPR2, was observed. This condition led to increased apoptosis and vascular remodeling in the lungs [34].

### 2.3 Serotonin Transporter

The serotonin transporter (*SERT*) gene plays a critical role in the genetic landscape of PH, affecting disease onset, severity, and progression through its promoter polymorphisms and pathway interactions. In both familial and idiopathic forms of PAH, the *SERT* promoter polymorphism modulates PH risk. The long (L) allele, linked to increased *SERT* transcription, is associated with earlier onset in familial PAH (FPAH), particularly among patients with *BMPR2* mutations, suggesting a genetic interaction between *SERT* and *BMPR2* that may accelerate PAH onset in FPAH [35]. However, while the L allele has been linked to FPAH onset, it has not shown a significant effect on survival rates in either FPAH or idiopathic PAH (IPAH) cases [35]. Furthermore, SERT polymorphisms are not associated with the risk of portopulmonary hypertension in patients with advanced liver disease [36]. Genetically, *SERT* contributes to PH progression by interacting with the platelet‐derived growth factor receptor β (PDGFRβ), which promotes PASMC proliferation. Experimental studies have shown that SERT transactivates PDGFRβ in serotonin‐stimulated PASMCs, indicating a genetic predisposition that impacts platelet‐derived growth factor (PDGF) signaling pathways in PH [37]. Inhibiting or genetically downregulating *SERT* attenuates PDGF‐stimulated PASMC proliferation, with evidence suggesting that the presence of the PDZ motif in SERT is essential for PDGF‐induced Akt phosphorylation, underscoring a specific genetic requirement for SERT's regulatory impact on PH [38]. SERT also appears to play a significant role in the higher prevalence of PAH observed in females [38]. In female SERT‐overexpressing (SERT+) mice, microarray and quantitative reverse transcription polymerase chain reaction (qRT‐PCR) analyses have shown upregulation of genes associated with PAH, including *CCAAT/enhancer binding protein β* (*CEBPβ*), *cytochrome P450 family 1 subfamily B member 1* (*CYP1B1*), and *Fos proto‐oncogene*, *AP‐1 transcription factor subunit* (*FOS*). Protein expression of these genes was also elevated, indicating a genetic predisposition toward PAH in females [39]. In PASMCs from IPAH patients, similar upregulation of *CEBPβ, CYP1B1*, and *FOS* was observed, suggesting parallels in human PAH. This sex‐related genetic susceptibility is further supported by findings that 17β‐estradiol promotes PAH development in female SERT+ mice through upregulation of SERT, tryptophan hydroxylase‐1 (TPH1), and the 5‐HT(1B) receptor. Ovariectomy or inhibition of TPH1 or the 5‐HT(1B) receptor significantly reduces this PAH phenotype, highlighting estrogen's genetic influence on serotonin pathways in PAH [39]. While studies on *SERT* allele distributions (LL, LS, SS) in PAH patients compared with the general population have shown no significant differences [40], a meta‐analysis suggests that the LL genotype in the *SERT* L/S polymorphism may increase the risk of developing IPAH, as individuals with this genotype are at higher risk than those with the SS genotype [41].

### 2.4 Aquaporin‐1

Research shows that aquaporin‐1 (*AQP1*) variants are present in two unrelated families with PAH, one hereditary and one associated with scleroderma, identified from a cohort of 300 patients [42]. A family affected by HPAH was identified, with all three siblings carrying a novel missense variant of *AQP1* (*c.273C>G; p.Ile91Met*) [43]. Whole‐genome sequencing in PAH patients identified rare variants in *AQP1*, along with *SOX17* (*SRY‐box transcription factor 17*) and *ATP13A3* (*ATPase 13A3*), suggesting a significant role for *AQP1* in PAH genetics. Familial segregation of *AQP1* mutations with PAH further underscores its involvement in the disease's molecular mechanisms [44]. In mice, *Aqp1* deficiency attenuates vascular remodeling in hypoxic PH by reducing abnormal proliferation and migration of PASMCs, while also protecting lung endothelial cells from apoptosis [45]. In hypoxic PH, AQP1 is expressed in PASMCs and plays a critical role in migration. Hypoxia selectively increases AQP1 protein levels and intracellular calcium concentration ([Ca^2^⁺]і) in PASMCs. Blockade of calcium entry and *AQP1* silencing inhibit this hypoxia‐induced migration, indicating that *AQP1*’s genetic expression and function are pivotal in PASMC responses to hypoxia in pulmonary vascular remodeling [46]. AQP1 is essential for proliferation and migration in PASMCs, with its C‐terminal tail being crucial for these processes. The EF‐hand motif is not required for AQP1's role in hypoxia‐induced responses in PASMCs [47]. In hypoxic PH, AQP1 enhances PASMC proliferation and migration by upregulating β‐catenin levels, which in turn increases the expression of β‐catenin targets like c‐Myc and cyclin D1. This mechanism requires the AQP1 COOH‐terminal tail, as deletion of this region abolishes the effects on β‐catenin and its target genes [48]. Additionally, increased expression of AQP1 in PASMCs from preclinical PH models contributes to apoptosis resistance by modulating B‐cell lymphoma 2 (Bcl‐2) and Bcl‐2‐associated X protein (BAX) expression ratios. Elevating AQP1 levels in control PASMCs also conferred resistance to apoptosis, indicating a crucial role for AQP1 in regulating cell fate in PH [49]. In a Taiwanese idiopathic and heritable PAH cohort, some patients carried *BMPR2* gene variants, while others had *AQP1* and other PAH‐related gene variants, suggesting a potential link between these genetic factors in the pathogenesis of PAH [50]. In PAH, silencing of *BMPR2* in human pulmonary microvascular endothelial cells leads to reduced AQP1 expression at the mRNA, protein, and functional levels. This finding suggests that decreased BMPR2 may contribute to AQP1 dysfunction, highlighting a potential mechanism involved in PAH pathogenesis [51]. Silencing the *AQP1* gene increased the expression of BMP9 and BMP10 in human pulmonary microvascular endothelial cells, indicating a regulatory role for AQP1 within the BMP signaling pathway. Furthermore, exogenous BMP9 administration restored decreased AQP1 protein levels, highlighting its potential therapeutic implications in PAH [52]

### 2.5 ATPase 13A3

A study identified ATPase 13A3 (*ATP13A3*) variants as one of the most frequent genetic contributors to PAH in Asian cohorts, alongside *BMPR2* and *growth differentiation factor 2* (*GDF2*) [53]. Rare variants in the *ATP13A3* gene have been validated as contributing to approximately 2.7% of PAH cases, alongside other channelopathy genes [54]. A multistep genetic screening identified *ATP13A3* as a novel candidate gene associated with PAH through its involvement in lung vascular remodeling [55]. Additionally, gene panel diagnostics identified disease‐causing variants in *ATP13A3* in families with at least two PAH patients [56]. Whole‐genome sequencing in PAH cases revealed a significant overrepresentation of rare variants in *ATP13A3*, suggesting its potential role in the disease's genetic basis [44]. In a study of childhood‐onset PAH, whole exome sequencing revealed a novel heterozygous mutation in the *ATP13A3* gene as a genetic risk factor [57]. In a case involving a 21‐month‐old patient, biallelic mutations in the *ATP13A3* gene were linked to malignant progression of PAH, prompting a high‐risk Potts shunt procedure [58]. Biallelic variants in the *ATP13A3* gene are associated with autosomal recessive childhood‐onset PAH, characterized by high mortality and treatment resistance. The findings suggest a likely semidominant, dose‐dependent inheritance for *ATP13A3* in PAH cases [59]. In a study of multiple sclerosis patients treated with interferon beta 1a (IFN‐β 1a), a nonsense variant in the *ATP13A3* gene was identified in one patient who developed PAH. This suggests that genetic predisposition involving ATP13A3 may increase the risk of PAH in patients undergoing IFN‐β 1a therapy, warranting further investigation [60]. ATP13A3 is identified as a crucial polyamine transporter in endothelial cells, where its deficiency leads to impaired polyamine homeostasis and endothelial dysfunction, contributing to PAH. Additionally, specific PAH‐associated *ATP13A3* variants demonstrate loss‐of‐function effects, and mice with an *ATP13A3* variant developed a PAH phenotype, highlighting its role in disease pathogenesis [61].

### 2.6 SRY‐Box Transcription Factor 17

*SOX17* mutations are significantly associated with PAH, showing familial segregation with the disease and suggesting a key role in its heritability [44, 62–64]. Pathogenic variants in *SOX17*, though less common than other genes associated with HPAH, have been identified in patients, highlighting its role in disease susceptibility [65]. A novel heterozygous mutation in *SOX17* (c.379C>T; p.Gln127*) was identified in a consanguineous family with autosomal‐dominant PAH, exhibiting complete penetrance and loss of function [66]. In a case study of four Japanese patients with PAH, *SOX17* mutations were identified, supporting SOX17 as a novel causative gene for PAH [67]. *SOX17* has been identified as a novel risk gene for PAH, with rare deleterious variants contributing to 3.2% of PAH cases with CHD and 0.7% of idiopathic/familial PAH cases. These variants, particularly in the conserved HMG‐box domain, implicate *SOX17* and its targets in the development of pulmonary vasculature, underscoring its importance in PAH pathogenesis [68, 69, 70]. Moreover, genome‐wide association studies identified a locus near *SOX17* associated with increased risk for PAH, showing enhancer activity that affects *SOX17* regulation in endothelial cells. Risk variants near *SOX17* indicate that impaired *SOX17* function is a significant factor in PAH, extending beyond rare mutations and impacting gene expression through common genetic variations [71, 72]. Another genome‐wide association study found that PAH‐related stimuli upregulate SOX17‐mediated NAMPT promoter activity in endothelial cells, which contributes to vascular remodeling in PAH [73]. SOX17 expression is significantly downregulated in patients with IPAH, and its deficiency in endothelial cells exacerbates PH via E2F1 (E2F transcription factor 1)‐mediated dysfunctions [74]. SOX17 deficiency is associated with increased PAH risk, with the genetic variant rs10103692 linked to reduced plasma citrate levels in PAH patients. Additionally, the downregulation of *SOX17* by the pathological estrogen metabolite 16α‐hydroxyestrone may contribute to PAH development, linking sexual dimorphism and *SOX17* genetics in PAH [75, 76]. In mice, *Sox17* deficiency in pulmonary endothelial cells promotes the development of PAH under hypoxia by enhancing hypermuscularization and inflammation, while also increasing hepatocyte growth factor/mesenchymal–epithelial transition factor signaling [77]. The biomimetic model of PAH reveals a critical BMPR2–SOX17–prostacyclin (PGI2) signaling axis, highlighting SOX17's role in endothelial‐smooth muscle cell interactions and the remodeling process [78]. A study identified SOX17 as a crucial transcription factor mediating BMP9‐induced *semaphorin 3G* expression, linking it to endothelial function and vascular regulation [79]. SOX17 plays a crucial role in maintaining endothelial function and vascular homeostasis in PH by regulating exosomal miRNAs that mitigate endothelial injury and promote vascular health [80]. Genetic risk variants upstream of the *SOX17* promoter impair transcription factor binding, leading to reduced SOX17 expression in human PAECs (HPAECs) and contributing to endothelial dysfunction in PAH. A study identified homeobox A5 (HOXA5) and retinoic acid receptor‐related orphan receptor‐α as key transcription factors involved, with potential drug compounds capable of reversing the negative transcriptomic effects associated with SOX17 dysregulation [81]. In pediatric‐onset PAH, rare deleterious variants in *SOX17* are significant genetic contributors, distinct from adult‐onset PAH, and associated with developmental origins of the disease [82, 83, 84, 85].

### 2.7 Kallikrein 1

Kallikrein 1 (*KLK1*) is identified as a novel candidate risk gene for IPAH, linked to a later mean age of onset and relatively moderate disease phenotypes. This discovery underscores *KLK1*’s role in vascular hemodynamics and inflammation, suggesting its potential as a therapeutic target in PAH [86]. However, *KLK1* is classified as having limited evidence for a causal relationship with PAH, suggesting that while it may contribute to the disease, more research is needed to establish its definitive role. Consequently, caution should be exercised in interpreting variants identified in *KLK1* during genetic testing for PAH [87].

### 2.8 Vascular Endothelial Growth Factor Receptor 2

While vascular endothelial growth factor receptor 2 (KDR) is expressed in endothelial cells within plexiform lesions in PH, indicating its role in the disease's pathophysiological changes [88], Fawn‐Hooded rats (FHR) raised at Denver's altitude showed comparable KDR expression to that of normal Sprague–Dawley and Fischer rat strains. This suggests that KDR is not directly implicated in the reduced pulmonary arterial density observed in this model of PH [89]. In previous studies, *KDR* was identified as a candidate gene associated with PAH, specifically IPAH based on the presence of rare deleterious variants [84, 87, 90]. A study identified heterozygous, high‐impact, likely loss‐of‐function variants in the *KDR* gene that were strongly associated with a reduced carbon monoxide transfer coefficient and an older age at diagnosis of PAH [91]. In mice, the lack of endothelial nitric oxide synthase (eNOS) and exposure to bleomycin led to decreased expression of phosphorylated VEGFR2 (KDR), suggesting that the disruption of the VEGF (vascular endothelial growth factor) signaling pathway contributes to PAH and impaired alveolar development. This highlights the importance of KDR in the vascular remodeling and pathophysiological processes associated with PH in the context of bronchopulmonary dysplasia [92]. In a study investigating the genetic basis of PAH, researchers found that mutations in the *KDR* gene were associated with severe PAH in two families, characterized by low diffusing capacity for carbon monoxide (D_LCOc) and interstitial lung disease. This evidence supports the classification of *KDR* as a newly identified gene implicated in the development of PAH, particularly in cases presenting with radiological lung parenchymal disease [93]. The conditional deletion of the *KDR* gene, which encodes VEGF receptor‐2, in mice, led to mild PH under normoxia that worsened under hypoxia, characterized by increased pulmonary arterial wall thickness and vessel occlusion [94].

### 2.9 T‐Box Transcription Factor 4

Studies identify T‐box transcription factor 4 (*TBX4*) as a potential risk gene for PH [95, 96, 97, 98, 99, 100, 101, 102, 103, 104, 105]. Research indicates that TBX4‐related PAH tends to have a milder progression, with late diagnosis being the sole predictor of negative outcomes in its hereditary forms [106]. In a case report, the identification of a microdeletion at 17q22q23.2 highlights the haploinsufficiency of the *TBX4* gene, which may play a significant role in the observed phenotypic features, particularly PH [107, 108]. A de novo missense mutation in the *TBX4* gene (*p.E86Q*) was identified in a deceased newborn with severe acinar dysplasia of the lungs, suggesting a novel association between *TBX4* mutations and this condition [109]. An intronic *TBX4* variant near the splice site of exon 3 led to significantly reduced TBX4 expression, associated with a spectrum of cardiopulmonary symptoms in affected family members. This case highlights the variable expressivity of *TBX4* mutations, linking them to both severe neonatal lung disease and milder adult phenotypes [110]. *TBX4* mutations were identified in a notable proportion of children with childhood‐onset PAH, while only a few mutations were found in adults with PAH, suggesting a stronger association between TBX4 and childhood‐onset PAH [85, 97, 111–116]. Additionally, many patients with *TBX4* mutations exhibited previously unrecognized small patella syndrome (SPS) [111]. Moreover, in a study of 40 children with IPAH or familial PAH, three mutations in the *TBX4* gene were identified, highlighting its role in the genetic architecture of pediatric PAH. *TBX4* mutations were associated with more severe disease at diagnosis, though the overall outcomes for mutation carriers were similar to those of noncarriers [117]. In a study, rare variants in the *TBX4* gene, including six deletions at the 17q23 locus and 12 likely damaging mutations, were identified as associated with a new form of developmental lung disease in children with PH. These variants contribute to severe, often biphasic PH, along with other congenital anomalies, highlighting TBX4's significant role in pediatric PH [118]. In another study, a paternally inherited heterozygous missense variant in the *TBX4* gene (*c.1198G>A, p.Glu400Lys*) was identified in a newborn with severe PH and other developmental abnormalities, suggesting its likely deleterious role. The findings indicate that *TBX4* variants, in conjunction with *CTNNB1* mutations, may synergistically contribute to a more severe phenotype in lethal lung developmental diseases [119]. In the case of a deceased newborn with PH and interstitial emphysema, two de novo heterozygous recurrent copy‐number variant deletions were identified, including one affecting the *TBX4* gene at 17q23.1q23.2. The study also detected seven novel regulatory noncoding single nucleotide variants upstream of *TBX4*, indicating a complex interplay between noncoding and coding variants that may influence lung development and contribute to lethal lung developmental disorders [120]. According to a study, the rs3744439 variant in the *TBX4* gene was significantly associated with susceptibility to PH, with its AG genotype exclusively found in healthy controls and absent in PH patients [121]. A study suggests that *TBX4*’s involvement alongside known PAH‐related genes like *BMPR2* indicates its potential significance in the genetic landscape of PAH in the Middle East and North Africa region [122]. Variants in the *TBX4* gene were identified as potential genetic causes of PAH in a subset of Japanese patients, disrupting its regulation of fibroblast growth factor (FGF) 10 and leading to insufficient lung morphogenesis. These findings suggest that *TBX4* variants contribute to PAH pathogenesis through impaired pulmonary vascular development and endothelial dysfunction [123]. A preclinical study found that *TBX4* is epigenetically reactivated in PAH, contributing to vascular remodeling, and silencing *TBX4* showed potential therapeutic effects in rodent models [124].

### 2.10 Eukaryotic Translation Initiation Factor 2 Alpha Kinase 4

Research highlights *TBX4* as a possible risk gene associated with PH [55, 125–132]. Recessive mutations in the eukaryotic translation initiation factor 2 alpha kinase 4 (*EIF2AK4*) gene, in either homozygous or compound‐heterozygous states, are strongly associated with PH, specifically in the context of pulmonary veno‐occlusive disease (PVOD), where they disrupt EIF2AK4 function and contribute to disease progression [133, 134]. PVOD is an uncommon cause of PAH that can be categorized as idiopathic, heritable, drug‐induced, radiation‐induced, or associated with connective tissue disorders [135]. A sporadic PVOD patient with confirmed biallelic *EIF2AK4* mutations displayed an atypical treatment response to PAH‐targeted drugs, suggesting genetic heterogeneity in PVOD [136]. Pulmonary capillary hemangiomatosis (PCH) is a rare condition marked by abnormal capillary growth in the alveolar interstitium, exhibiting similarities to PVOD, both of which are associated with PH [137]. In a whole‐exome sequencing study, two novel pathogenic variants in the *EIF2AK4* gene (*c.2137_2138dup* and *c.3358‐1G>A*) were identified in a patient diagnosed with PVOD/PCH [138]. While studies have associated *EIF2AK4* genetic mutations with PVOD/PCH [139, 140, 141, 142, 143, 144, 145, 146, 147, 148], in a large cohort study, biallelic *EIF2AK4* mutations were identified in patients clinically diagnosed with idiopathic and heritable PAH, suggesting a misclassification of some cases that resemble PVOD/PCH. These patients exhibited distinct characteristics, such as a low carbon monoxide transfer coefficient (Kco) and earlier onset, underscoring the value of genetic testing for accurate diagnosis and management [149]. In a study, bi‐allelic mutations in the *EIF2AK4* gene were identified, including a novel nonsense mutation (*c.1672C>T*) and a documented deletion (*c.560_564drlAAGAA*), in two nonconsanguineous families with PH. These findings underscore the involvement of *EIF2AK4* mutations in the diverse phenotypic expressions of PH, with differing clinical outcomes observed between symptomatic and asymptomatic individuals [150]. In a semiconductor chip‐based next‐generation sequencing study of PH patients, a homozygous *EIF2AK4* variant (*p.P1115L*) was identified, highlighting its role in PH genetic susceptibility [151]. Also, in Iberian Gypsy families with severe PAH, homozygous *EIF2AK4* mutation (*c.3344C>T, p.P1115L*) was identified, showing an aggressive phenotype and necessitating early lung transplantation [152]. In a study, a novel *EIF2AK4* splice site mutation was identified alongside a *BMPR2* mutation in family members with autosomal dominantly inherited HPAH, supporting a “second hit” model. The presence of both mutations led to manifest HPAH, suggesting EIF2AK4 as a potential modifier of PAH risk in conjunction with BMPR2 [153]. A study showed that the *EIF2AK4* gene encodes general control nonderepressible 2 kinase (GCN2), which mediates pulmonary vascular remodeling and PAH. Notably, loss of GCN2 activity did not induce PVOD or PAH in mice, suggesting a nuanced role for *EIF2AK4* in these conditions. GCN2 activation was linked to hypoxia‐induced endothelin‐1 expression, indicating its potential as a therapeutic target in PAH without *EIF2AK4* mutations [154].

### 2.11 ATP Binding Cassette Subfamily C Member 8

ATP binding cassette subfamily C member 8 (*ABCC8*) is a member of the C branch of the ABC transporter superfamily, encoding sulfonylurea receptor 1 (SUR1), an ATP‐sensitive potassium channel regulatory subunit [54, 62]. *ABCC8* is identified as one of the most common causes of PAH, accounting for approximately 1% of cases [54]. In the US‐PAH biorepository, which includes 2572 PAH cases subjected to whole exome sequencing, predicted deleterious missense variants in *ABCC8* were identified in 28 patients [86]. The International PH Expert Panel employed a semi‐quantitative scoring system developed through the NIH Clinical Genome Resource to classify the relative strength of evidence supporting gene‐disease relationships for PAH based on genetic and experimental data, *ABCC8* was categorized as having moderate evidence of clinical relevance to PAH [87].

Through exome sequencing of 99 pediatric and 134 adult PAH cases, 11 novel or rare missense mutations located in conserved regions of *ABCC8* were identified*: c.A214G (p.N72D)*, *c.G558T (p.E186D), c.G718A (p.A240T), c.G2371C (p.E791Q), c.T2694+2G, c.G331A (p.G111R), c.C403G (p.L135* *V), c.G2437A (p.D813N), c.G4414A (p.D1472N), c.C686T (p.T229I)*, and *c.G3941A (p.R1314H)*. These mutations contribute to the loss of K_ATP channel functionality and are associated with PAH [155]. Additionally, genetic sequencing of 624 PAH cases from the Spanish national registry revealed 11 further variants in the *ABCC8* gene [156]. Following computational and in vitro biochemical analyses, Lago‐Docampo et al. [156] classified two variants as pathogenic (*c.3288_3289del* and *c.3394G>A*), six as likely pathogenic (*c.211C>T, c.1429G>A, c.1643C>T, c.2422C>A, c.2694+1G>A*, and *c.3976G>A*), and three as variants of unknown significance (*c.298G>A, c.2176G>A*, and *c.3238G>A*). A novel heterozygous missense variant 21 was observed in a 19‐month‐old male patient with PAH [57].

Mutations in *ABCC8* may impair the K_ATP‐independent effects of SUR1 that induce cellular apoptosis, potentially promoting the intimal proliferation or excessive growth of the intima observed in PAH. Pharmacological activation of SUR1 using three distinct activators: diazoxide, VU0071063, and NN414 results in vasorelaxation of pulmonary arteries, while inhibition of the SUR1/Kir6.2 channel is associated with vasoconstriction [157]. Moreover, metformin has been shown to selectively activate *ABCC8*, thereby restoring channel function [158].

### 2.12 Gamma‐Glutamyl Carboxylase

Gamma‐glutamyl carboxylase (GGCX), also known as vitamin K‐dependent gamma‐carboxylase, plays a critical role in the vitamin K cycle by catalyzing the gamma‐carboxylation of glutamic acid residues in vitamin K‐dependent proteins [159]. *GGCX* is involved in various biological processes, including inflammation, bone metabolism, blood coagulation, vascular calcification, and cell proliferation [160, 161, 162]. Recent studies have suggested a potential link between *GGCX* and PAH (Table 1). Mutations in *GGCX* have been classified by an international expert panel on PAH as having moderate evidence of clinical relevance to the disease [87]. Notably, Zhu et al. [86] conducted whole exome sequencing of 2572 PAH patients from a national biorepository, identifying 28 cases with *GGCX* variants. They hypothesized that these mutations may alter the inflammatory responses or cell proliferation in PASMCs or PAECs, both of which are hallmarks of PAH [86].

**TABLE 1:** The role of genes in PH.

Gene symbol	Cell/tissue	Expressions	Function	References
*BMPR2*	PASMCs	Downregulation	Proliferation, reduce contractility, stimulate glucose metabolism	[24, 25, 27]
	Human PAECs	Downregulation	Proliferation, reduce NK cell numbers	[23, 26]
*Cav‐1*	PASMCs	Downregulation	Diminish vasodilation, and apoptosis, inhibit the proliferation of PASMCs, vascular remodeling	[30, 31, 33]
	Human PAECs	Downregulation	Vascular remodeling	[32, 34]
*SERT*	PASMCs	Upregulation	Proliferation	[37]
	PASMCs	Downregulation	Attenuate proliferation	[38]
*AQP1*	PASMCs	Downregulation	Attenuate proliferation, attenuate vascular remodeling, protect lung endothelial cells from apoptosis	[45]
	PASMCs	Upregulation	Enhance PASMC proliferation and migration	[48]
	PASMCs	Upregulation	Apoptosis resistance	[49]
*ATP13A3*	Plasma	Mutation	Lung vascular remodeling	[55]
	Human PAECs	Deficiency	Impaired polyamine homeostasis, endothelial dysfunction	[61]
*SOX17*	Endothelial cells	Upregulation	Vascular remodeling	[73]
	Endothelial cells	Downregulation	Exacerbates PH via E2F1‐mediated dysfunctions	[74]
	PAECs	Deficiency	Enhance hypermuscularization and inflammation	[77]
	Human PAECs	Downregulation	Remodel pulmonary artery endothelium	[80]
	Human PAECs	Variant	Endothelial dysfunction	[81]
*KLK1*	Plasma	Variant	Vascular hemodynamics, inflammation	[86]
*KDR*	Lung	Downregulation	Impaired alveolar development	[92]
	Human PAECs	Mutation [*KDR* mutation (*c.976+2T>C*), *KDR* truncating mutation (c.3302T>G, p.Leu1101*)]	Low diffusing capacity for carbon monoxide, interstitial lung disease	[93]
	Murine PAECs	Conditional deletion	Increased pulmonary arterial wall thickness, vessel occlusion	[94]
*TBX4*	Peripheral blood, lung	Mutation [*TBX4* missense mutation *p.E86Q (c.256G>C)*]	Acinar dysplasia of the lungs	[109]
	Lung	Mutation	Small patella syndrome (SPS)	[111]
	Plasma, lung	Variant [*TBX4* variant (*c.1198G>A, p.Glu400Lys*)]	lethal lung developmental diseases	[119]
	Plasma, lung	Variant	Influence lung development, contribute to lethal lung developmental disorders	[120]
	Peripheral blood leukocyte	Variant	Insufficient lung morphogenesis, impaired pulmonary vascular development, endothelial dysfunction	[123]
	PAFs	Reexpressed	Vascular remodeling	[124]
*EIF2AK4*	Lung	Mutation [*EIF2AK4* mutation (*c.2137_2138dup and c.3358‐1G>A*)]	PVOD, PCH	[138]
	Plasma	Mutation	Manifest HPAH	[153]
*ABCC8*	Human PAECs, SMCs	Mutation	Loss of K_ATP channel functionality, intimal proliferation, excessive growth of the intima	[157]
	Human PAECs	Inhibition	Vasoconstriction	[157]
*GGCX*	Plasma	Mutation	Alter the inflammatory responses or cell proliferation in PASMCs or PAECs	[86]

Abbreviations: PH, pulmonary hypertension; PASMCs, pulmonary artery smooth muscle cells; PAECs, pulmonary artery endothelial cells; PAFs, pulmonary arterial fibroblasts; SMCs, smooth muscle cells; NK cell, natural killer cell; HPAH, heritable pulmonary arterial hypertension; PVOD, pulmonary veno‐occlusive disease; PCH, pulmonary capillary hemangiomatosis.

## 3 Epigenetic Mechanisms of Pathogenesis in PH

Epigenetics, the study of inheritable changes in phenotype and gene expression that occur without alterations to the DNA sequence, has emerged as a crucial factor in disease pathogenesis. These changes, known as epigenetic marks, are shaped by interactions between the genome and environmental influences, and they modify chromatin structure to regulate gene expression through mechanisms such as RNA and DNA methylation, histone modifications, and ncRNAs [163, 164, 165, 166, 167, 168, 169, 170, 171]. While epigenetic modifications can promote genetic diversity, they often lead to developmental disorders and diseases. Disruptions in these regulatory mechanisms are linked to a variety of conditions, including autoimmune diseases, cancer, and PH [164, 165, 169, 172–174]. Although much of the research on epigenetics has focused on cancer and other well‐known diseases, the role of epigenetic dysregulation in the development of PAH is now gaining increasing attention.

### 3.1 RNA Methylation

**FIGURE 3:**
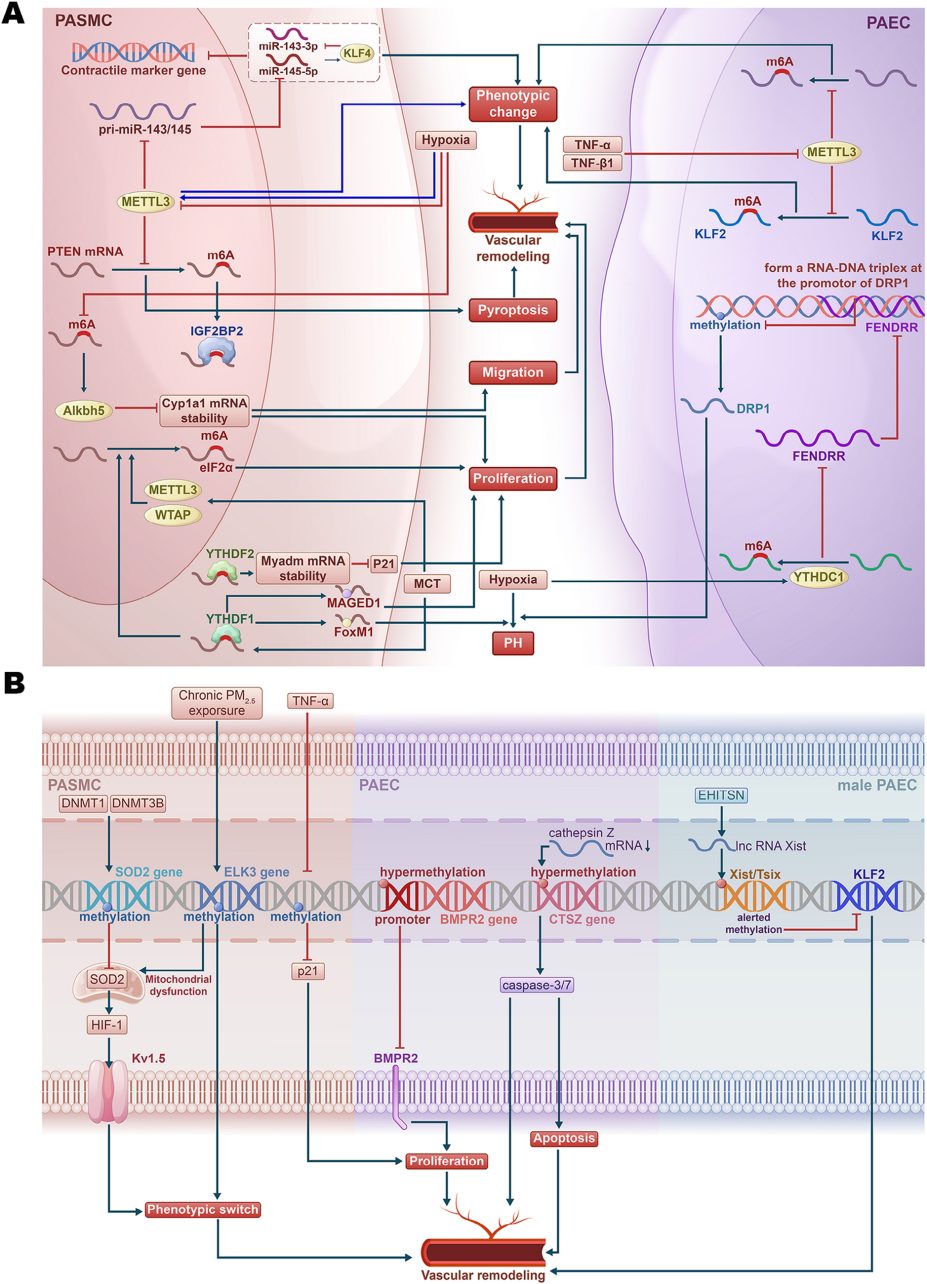
The role of RNA methylation, DNA methylation, and demethylation in PH. (A) RNA methylation primarily affects RNA stability and gene expression. In PASMCs, hypoxia‐induced reduction of METTL3 inhibits m6A methylation, leading to cellular phenotypic shifts and pyroptosis. Additionally, hypoxia increases Alkbh5, which promotes cell migration and proliferation by destabilizing Cyp1a1 mRNA, contributing to PH. However, some studies also suggest that hypoxia‐induced upregulation of METTL3 can drive phenotypic changes. In MCT‐induced PH, elevated YTHDF1 enhances the translation of MAGED1 and FoxM1, accelerating disease progression, while YTHDF2 upregulation in PASMCs promotes proliferation via the m6A/Myadm/p21 pathway. In PAECs, TNF‐α and TGF‐β1 suppress METTL3, reducing RNA methylation and leading to vascular remodeling and phenotypic changes. Furthermore, hypoxia‐induced inhibition of FENDRR by YTHDC1 decreases DRP1 promoter methylation, promoting DRP1 expression and contributing to PH development. (B) DNA methylation and demethylation regulate key gene expression, influencing cell proliferation, phenotypic switching, and vascular remodeling. In PASMCs, methylation of the SOD2 gene suppresses its expression, leading to HIF‐1 activation and phenotypic changes, while methylation of the ELK3 gene induces mitochondrial dysfunction, further promoting cellular shifts that contribute to PH. In PAECs, hypermethylation of the BMPR2 promoter and the CTSZ gene impairs BMPR2 activity and apoptosis, facilitating vascular remodeling and disease progression. Additionally, in male PAECs, EHITSN upregulates Xist expression, altering the methylation of the Xist/Tsix locus, which reduces KLF2 expression and drives vascular remodeling, ultimately leading to PH. PASMC, pulmonary artery smooth muscle cell; PAEC, pulmonary artery endothelial cell; m6A, N6‐methyladenosine; pri‐miR, primary microRNA; PH, pulmonary hypertension; PM, particulate matter.

Recent studies have underscored the pivotal role of RNA methylation, specifically N6‐methyladenosine (m6A), in PH pathogenesis (Figure 3A) [175, 176, 177, 178, 179]. This type of methylation plays a crucial role in regulating mRNA stability, translation, and degradation, influencing various cellular processes, including proliferation, apoptosis, pyroptosis, and EndMT in the context of PH. Using bioinformatics analysis, Gao et al. [175] identified key m6A RNA methylation regulators, revealing that fragile X messenger ribonucleoprotein 1 and heterogeneous nuclear ribonucleoprotein (hnRNP) A2/B1 are upregulated in IPAH, while leucine‐rich pentatricopeptide repeat containing is downregulated. Experimental validation in human PASMCs (HPASMCs) indicates that m6A modification may significantly influence the immune microenvironment and progression of IPAH [175].

While RNA methyltransferase methyltransferase 3 (METTL3) is upregulated in response to hypoxia, promoting vascular remodeling by influencing the phenotypic switching of PASMCs [180, 181], research demonstrates that low sustained expression of METTL3 also facilitates PH by affecting the m6A modification of PH‐related genes [181, 182, 183, 184]. For instance, targeted deletion of METTL3 exacerbates hypoxia‐induced PH and enhances PASMC phenotypic switching through decreased m6A modification and impaired processing of pri‐miR‐143/145. This leads to reduced levels of miR‐143‐3p and miR‐145‐5p, resulting in the upregulation of Krüppel‐like factor 4 (KLF4), which further suppresses miR‐143/145 transcription. This positive feedback loop sustains the contractile marker gene suppression and PASMC phenotype changes [181]. Additionally, in HPAECs, the global levels of m6A and METTL3 expression decrease during TNF‐α‐ and TGF‐β1‐induced EndMT, promoting a shift toward mesenchymal markers and exacerbating pulmonary vascular remodeling and hypoxia‐induced PH in mice. METTL3‐mediated m6A modification of KLF2 is critical for maintaining endothelial characteristics; its overexpression counteracts EndMT by upregulating endothelial markers and downregulating mesenchymal markers, while mutations in the m6A site of *KLF2* mRNA impair its protective effects [183]. Jiang et al. [184] showed that the downregulation of METTL3 in PH leads to decreased m6A methylation, promoting pyroptosis in PASMCs. Mechanistically, METTL3 modulates the stability of phosphatase and tensin homolog (PTEN) mRNA through m6A modification and interaction with the m6A reader protein insulin‐like growth factor 2 mRNA‐binding protein 2 [184].

The m6A reader protein YTH N6‐methyladenosine RNA binding protein F1 (YTHDF1) promotes PH through the regulation of MAGE family member D (MAGED1) translation in an m6A‐dependent manner [185]. Another study showed that YTHDF1 enhances Forkhead box M1 (FoxM1) translation efficiency, further driving hypoxia‐induced PH [186]. Moreover, YTH N6‐methyladenosine RNA binding protein C1 (YTHDC1)‐mediated m6A modifications were found to induce the degradation of the lncRNA FOXF1 adjacent noncoding developmental regulatory RNA (FENDRR), a process that contributes to hypoxia‐induced PH. FENDRR plays a key regulatory role by forming an RNA–DNA triplex at the promoter of *dynamin‐related protein 1* (*DRP1*), increasing promoter methylation and thereby reducing *DRP1* transcription [187]. Additionally, YTHDF2 is upregulated in PASMCs during PH and promotes cell proliferation by stabilizing myeloid‐associated differentiation marker (MYADM) mRNA in an m6A‐dependent manner, while also inhibiting cyclin‐dependent kinase inhibitor 1A (p21) [188]. In hypoxia‐induced PH, the upregulation of AlkB homolog 5 (Alkbh5) is associated with a decrease in m6A modifications, affecting the stability of cytochrome P450 family 1 subfamily A member (CYP1A1) mRNA, and consequently impacting PASMC proliferation and migration [189].

Recent findings indicate that eukaryotic initiation factor 2α (eIF2α) is significantly upregulated in monocrotaline (MCT)‐PAH rats, with its expression linked to m6A methylation. Elevated levels of METTL3, WT1‐associated protein (WTAP), and YTHDF1 contribute to this m6A modification, promoting PASMC proliferation and exacerbating PAH. The eIF2α inhibitor GSK2606414 can reduce eIF2α expression and alleviate PASMC proliferation, suggesting a novel regulatory mechanism where m6A modification of eIF2α plays a critical role in PAH pathogenesis [190].

These mechanisms highlight how m6A‐mediated RNA methylation promotes PH development by influencing the epigenetic regulation of critical genes under hypoxic conditions, suggesting that m6A modifications play a crucial role in modulating PH pathogenesis, particularly through hypoxia‐related pathways.

### 3.2 DNA Methylation and Demethylation

DNA methylation is a vital epigenetic mechanism that plays a crucial role in regulating gene expression by adding a methyl group to cytosine residues within CpG dinucleotides. This process is primarily mediated by DNA methyltransferases (DNMTs), which control gene activity by either silencing or activating specific genetic regions. In mammalian cells, this process contributes to gene repression via various pathways, including hindering transcription factor binding, recruiting methyl‐CpG binding proteins that interfere with transcriptional regulators, or altering chromatin structure to limit DNA accessibility. While the majority of CpG sites in healthy somatic cells are methylated, promoter regions, particularly CpG islands, tend to be protected from this modification [191, 192, 193, 194]. On the other hand, DNA demethylation is the process of removing these methyl groups, which can lead to the reactivation of silenced genes. Demethylation can occur through passive mechanisms, such as during DNA replication when methylation patterns are not copied, or through active mechanisms involving specific enzymes like TET (ten‐eleven translocation) proteins, which convert 5‐methylcytosine to 5‐hydroxymethylcytosine, eventually leading to complete demethylation [195, 196, 197]. Together, DNA methylation and demethylation are crucial in physiological processes such as embryonic development, genomic imprinting, and X‐chromosome inactivation. However, aberrant methylation patterns, whether through hypermethylation or hypomethylation, have been implicated in a growing number of diseases, including PH. Emerging evidence suggests that dysregulated DNA methylation contributes to PH pathogenesis (Figure 3B).

Reduced representation bisulfite sequencing identified 631 differentially methylated CpG sites in circulating CD4^+^ T cells from PAH patients, highlighting the *suppressor of cytokine signaling 3* as a key gene with hypomethylation associated with adverse hemodynamic parameters [198]. In human studies, DNA methylation patterns have been shown to differentiate between PVOD and PAH. For instance, increased methylation of the *granulysin* gene was observed in patients with PVOD but not in those with PAH, suggesting that DNA methylation signatures could serve as potential biomarkers for distinguishing between these two conditions [199]. In a study investigating PAH, it was found that DNA methylation significantly influences the expression of superoxide dismutase (SOD) 2, a key antioxidant enzyme located in mitochondria. The research demonstrated that levels of SOD2 are significantly reduced in PASMCs taken from individuals with PAH. It also showed similar findings in FHR, a strain known to be susceptible to spontaneous PAH, which serves as a valuable model for studying the disease [200, 201]. The reduction in SOD2 expression leads to increased activation of hypoxia‐inducible factor‐1 (HIF‐1), resulting in elevated expression of voltage‐gated potassium channels (Kv1.5), which are sensitive to oxygen levels. This cascade of changes impairs oxygen sensing and negatively affects redox status in both the cytoplasm and mitochondria [200]. Additionally, the suppression of *SOD2* transcription and translation using small interfering RNA (siRNA) in normal PASMCs from Sprague–Dawley rats produces phenotypic features characteristic of PAH. Conversely, restoring SOD2 expression diminishes HIF‐1 levels and reinstates Kv1.5 expression in PASMCs from FHR [201]. Genomic bisulfite sequencing has identified a specific hypermethylated CpG island in the promoter and enhancer regions of the *SOD2* gene [201]. Notably, elevated DNA methylation levels correlate with increased expression of DNMT1 and DNMT3B in PASMCs from both PAH patients and FHR. Moreover, employing 5‐aza‐2'‐deoxycytidine, a DNMT inhibitor, effectively reduces DNA methylation, thereby restoring SOD2 levels, decreasing cell proliferation, and promoting programmed cell death in PASMCs derived from FHR [201].

HPAH, which accounts for 25–30% of PAH cases, further underscores the role of DNA methylation in disease development [202]. Genetic studies have identified causal mutations in several genes associated with PAH, including the bone *BMPR2*, *TBX4*, and others [202]. Among these, mutations in the *BMPR2* gene are recognized as significant risk factors for PAH, with a female penetrance of 42% and a male penetrance of 14% [125]. BMPR2 is crucial in the TGF‐β signaling pathway, which is involved in cellular differentiation and bone formation [203]. Interestingly, Liu et al. [204] demonstrated that hypermethylation of the *BMPR2* promoter leads to decreased expression of BMPR2 in patients with HPAH. This reduction in BMPR2 levels in PAECs promotes cell proliferation and vascular remodeling, which are key drivers of PAH progression. However, contrasting findings were reported by Poussada et al. [205], who found no evidence of *BMPR2* methylation in PAH patients when investigating the methylation patterns of the *BMPR2* promoter region in genomic DNA isolated from peripheral blood. These discrepancies may arise from variations in patient populations and the specific regions of the *BMPR2* gene analyzed [17, 204, 205]. In an epigenome‐wide association study, Ulrich et al. identified significant DNA methylation changes in PAH, highlighting three hypermethylated loci: *cathepsin Z* (*CTSZ)*, *component of oligomeric Golgi complex 6*, and *zinc finger protein 678*, with *CTSZ* showing a correlation to reduced cathepsin Z mRNA levels [206]. Hypermethylation in *CTSZ* was associated with increased caspase‐3/7 activity, implicating its role in endothelial apoptosis and pulmonary vascular remodeling. These findings suggest that epigenetic alterations, particularly in *CTSZ*, contribute to the molecular mechanisms underlying PAH, alongside the limited involvement of established PAH genes like BMP10 [206]. Fine particulate matter (PM_2.5_) as an environmental pollutant is related to respiratory and cardiovascular diseases [207]. Chronic PM_2.5_ exposure has been shown to induce PAH by causing mitochondrial dysfunction and phenotypic switching in HPASMCs, with DNA methylation affecting the hub gene *ETS transcription factor ELK3* (*ELK3*) [207].

In a study, Yang et al. [208] demonstrated that global hypomethylation is linked to the loss of p21, a cyclin‐dependent kinase inhibitor, in the pulmonary vasculature of fetal lambs exposed to long‐term hypoxia at high altitudes. This hypomethylation‐induced reduction in p21 may trigger excessive proliferation of PASMCs, contributing to pulmonary vascular remodeling and the development of PH in these newborn lambs [208]. Additionally, DNA methylation was reduced in the first intron of the *endothelin‐1* (*ET‐1*) gene in pulmonary vascular endothelial cells (PVECs) and sperm of the first generation and also in the PVECs of the second generation. This demethylation facilitated the intergenerational transmission of endothelial dysfunction, perpetuating the PAH‐like features in offspring [209]. TNF‐α has been shown to significantly decrease DNA methylation in PASMCs without altering DNMT1 activity. The increased expression of growth arrest and DNA damage‐inducible α suggests a role in mediating DNA demethylation, which ultimately leads to increased PASMC proliferation. This effect was reversible with a superoxide scavenger (tempol), linking oxidative stress to DNA hypomethylation and subsequent cellular effects [210].

Furthermore, the epigenetic modification of X chromosome inactivation in females likely impacts the expression of PAH‐related genes, potentially contributing to the observed sex differences in the disease [211, 212]. Carman et al. [213] demonstrated that increased expression of the lncRNA X‐inactive‐specific transcript (Xist) in male PAECs is linked to altered DNA methylation at the *Xist/Tsix* (*TSIX transcript, XIST antisense RNA*) locus, contributing to PAH. This upregulation of Xist, driven by an intersectin‐1s protein fragment (EHITSN), leads to decreased expression of the protective gene *KLF2*, suggesting that Xist‐mediated changes in DNA methylation may play a critical role in the sex bias and pathogenesis of PAH in males [213].

Overall, DNA methylation and demethylation are crucial epigenetic mechanisms affecting both sporadic and hereditary forms of PH. Hypermethylation of protective genes leads to their silencing, resulting in increased oxidative stress, cell proliferation, and in some cases, apoptosis. Similarly, hypomethylation of key regulatory genes also contributes to these pathological effects. This dual role of methylation in promoting both abnormal cell growth and death underscores the potential for targeting epigenetic modifications as therapeutic strategies in PAH. Further research into these methylation patterns in diverse patient populations is essential for advancing personalized epigenetic therapies.

### 3.3 Histone PTMs

Histones are highly conserved proteins that serve as the primary constituents of chromatin structure. The histone octamer, composed of core histone dimers including H2A, H2B, H3, and H4 encases genomic DNA, forming nucleosomes that represent the basic repeating units of chromatin [214, 215]. The arrangement of DNA around this octamer influences chromatin structure and gene expression. PTMs of histones are fundamental in regulating gene expression by either promoting or inhibiting transcriptional activity. From a chemical perspective, histone modifications can be categorized into two main groups: one involving small organic substituents (acetylation, methylation, phosphorylation, deamination, and palmitoylation), and the other involving larger organic molecules (ubiquitylation, SUMOylation, biotinylation, glycosylation, and ADP‐ribosylation) [216, 217]. While the most extensively studied PTMs include acetylation and methylation, several lesser‐known modifications, such as glycosylation, biotinylation, SUMOylation, phosphorylation, ADP‐ribosylation, and ubiquitination, also contribute to chromatin dynamic [218]. Recent studies have introduced a range of acylations such as propionylation, butyrylation, crotonylation, succinylation, malonylation, and 2‐hydroxyisobutyrylation identified through mass spectrometry. These novel modifications provide a broader biochemical context that can influence gene promoters and transcriptional regions, similar to traditional acetylation [219].

Histone methylation and acetylation involve the addition of acetyl or methyl groups to specific lysine and/or arginine residues within the N‐terminal domain, often referred to as the histone “tail,” which extends from the nucleosomal surface. This region is critical for modulating chromatin folding and the recruitment of regulatory proteins [219, 220].

Evidence indicates that PTMs play a crucial role in the pathogenesis of PH (Table 2) [17, 221–223]. Understanding these modifications is vital, as alterations in histone modifications may influence gene expression patterns associated with the disease, thereby offering potential avenues for therapeutic intervention.

**TABLE 2:** The role of histone posttranslational modifications in PH.

Histone/enzyme	Activity	Targeted gene	Modification	Cell/tissue	Disease/model	Effects	References
SMYD2	Upregulation	*PPARγ*	Methylation	PASMC	Hypoxia‐PH	Vascular remodeling	[225]
H3K4(COMPASS)	Upregulation	*ICAMs, VCAMs*	Methylation	HUVEC, lung	Hypoxia‐PH	Leukocyte adhesion to endothelial cells, inflammation	[226]
H3K9 (G9a)	Upregulation	Global	Demethylation	PASMC	Inhibition of G9a by BIX‐01294	Proliferation, migration, contractility	[227]
H3K27(EZH2)	Upregulation	*Calponin*	Methylation	PASMC	Serum starved, hypoxia‐PH	Proliferation, migration, antiapoptosis, vascular remodeling	[228]
H3K27(EZH2)	Upregulation	*SOD1*	Methylation	Lung	TAC‐IPAH	Oxidative reactions	[229]
H3K4	Upregulation	*ET‐1*	Methylation	PVEC	Hypoxia‐induced second generations of IUGR rats	Proliferation, migration	[209]
H3K4/K9(LSD1)	Downregulation	*eNOS*	Demethylation	PASMC	Heterozygous knockout of *LSD1* mice	Proliferation, vascular abnormalities	[231]
H3/H4	Upregulation	*eNOS*	Acetylation	PVEC	PPHN		[237]
SIRT1	Downregulation	*Forkhead box protein O1/H1*	Acetylation	PASMC	SIRT1 pharmacological inhibition	Proliferation	[240]
HDAC1	Upregulation	*CCND1, COL1A1, MYLK, MCP1, Bcl2, survivin*	Deacetylation	PAAF, lung, PH‐Fib, R‐Cell	IPAH patients, MCT/hypoxia‐PAH, hypoxia‐PH	Proliferation, hypertensive phenotypes, apoptosis, inflammation	[239, 242, 244]
HDAC2	Upregulation	*YAP1, LEF1, CCND1, COL1A1, MYLK, MCP1, Bcl2*	Deacetylation	PAAF, lung	IPAH patients, MCT/hypoxia‐PAH	Proliferation, hypertensive phenotypes, apoptosis, inflammation	[239, 242]
HDAC3	Upregulation	*MCP1, Bcl2, SOD3*	Deacetylation	Lung, PASMC	IPAH patients, MCT/hypoxia‐PAH	Proliferation, inflammation	[239, 241]
HDAC5	Upregulation	*Survivin*	Deacetylation	PH‐Fib, R‐Cell	IPAH Patients, hypoxia‐PH	Proliferation	[244]
HDAC6	Upregulation	*Ku70*	Deacetylation	PASMC	IPAH patients, Su/Hx and MCT‐PAH	Proliferation, antiapoptosis, migration	[243]
HDAC8	Upregulation	*CCND1, COL1A1, MYLK*	Deacetylation	PAF	IPAH patients	Proliferation, hypertensive phenotypes, apoptosis	[242]
HDAC10	Downregulation	*NF‐κB*	Acetylation	PASMC	Cigarette smoke‐PH	Proliferation, antiapoptosis	[236]

Abbreviations: PASMC, pulmonary artery smooth muscle cells; HUVEC, human umbilical vein endothelial cell; PVEC, pulmonary vascular endothelial cell; PAAF, pulmonary arterial adventitial fibroblast; PAFs, pulmonary arterial fibroblasts; PH, pulmonary hypertension; TAC, transverse aortic constriction; IUGR, intrauterine growth restriction; PPHN, persistent pulmonary hypertension of the newborn; MCT, monocrotaline; Su/Hx, Sugen 5416/hypoxia; R‐Cell, rhomboidal cell; PH‐Fib, adventitial fibroblasts.

#### 3.3.1 Histone Methylation and Demethylation

Histone methylation is a crucial epigenetic modification involved in the regulation of gene expression, and it plays a significant role in the pathophysiology of PH (Table 2). This process involves the addition or removal of methyl groups on specific lysine and arginine residues of histone proteins, orchestrated by histone methyltransferases and histone demethylases (HDMs), respectively. The dynamic nature of histone methylation allows for tight regulation of gene transcription, which is particularly important in diseases like PH where altered gene expression contributes to vascular remodeling and inflammation. Two evolutionarily conserved families of HDMs, the lysine‐specific demethylases (LSDs) and the Jumonji C (JmjC) family, play key roles in histone demethylation. LSDs require flavin adenine dinucleotide (FAD) as a cofactor, whereas the JmjC family utilizes Fe^2^⁺ and α‐ketoglutarate for their enzymatic activity [217, 224]. Histone methylation can occur in mono‐, di‐, or trimethylation forms on lysine residues, while arginines can be monomethylated, symmetrically dimethylated, or asymmetrically dimethylated [220].

A study demonstrated that lysine methyltransferase SET and MYND domain containing 2 (SMYD2) is upregulated in PASMCs during PH and promotes cell proliferation by monomethylating PPARγ, which inhibits its activity. Inhibition of SMYD2 alleviates hypoxia‐induced pulmonary vascular remodeling, highlighting its role in PH development [225]. In PAH, histone methylation, particularly on lysine residues of histone H3, has been linked to altered vascular cell behavior and inflammation. The trimethylation of histone H3 on lysine 4 (H3K4me3) is associated with transcriptional activation. For instance, H3K4me3 facilitates the upregulation of cell adhesion molecules (CAMs), such as intercellular adhesion molecules (ICAMs) and vascular cell adhesion molecules (VCAMs), which promote leukocyte adhesion to endothelial cells and contribute to the inflammatory response seen in PAH. The myocardin‐related transcription factor A has been shown to interact with nuclear factor kappa B (NF‐κB), recruiting H3K4 methyltransferases to *CAM* promoters, thus driving CAM expression during hypoxic stress in PAH [226]. Downregulation of ASH2 and WD repeat domain 5 (WDR5), two key components of the H3K4 methyltransferase complex, significantly reduces CAM expression and ameliorates hypoxia‐induced PAH in mice, highlighting the therapeutic potential of targeting this pathway [226]. On the other hand, trimethylation of histone H3 on lysine 9 (H3K9me3) is typically associated with transcriptional repression. G9a (euchromatic histone lysine methyltransferase 2), an H3K9 methyltransferase, has been implicated in the proliferation, migration, and contractility of PASMCs in ovine fetal models of PH. Inhibition of G9a using BIX‐01294 has been shown to reduce these pathological processes, suggesting that H3K9 methylation plays a critical role in PASMC dysfunction in PH [227]. Additionally, the enhancer of zeste homolog 2 (EZH2), a key component of the polycomb repressive complex 2 responsible for H3K27 trimethylation (H3K27me3), has been found to be upregulated in PASMCs isolated from PAH animal models. This upregulation promotes PASMC proliferation, migration, and resistance to apoptosis, thereby contributing to vascular remodeling and disease progression [228]. Pharmacological inhibition of EZH2 using EPZ005687 has been shown to reduce reactive oxygen species (ROS) generation and reverse H3K27me3‐mediated repression of SOD1 in the pulmonary vasculature, improving outcomes in models of PAH [229]. In the first generation of intrauterine growth restriction (IUGR), trimethylation of H3K4me3 was significantly enhanced in PVECs and sperms. This histone modification contributed to the dysregulation of *ET‐1* gene expression, promoting endothelial dysfunction and susceptibility to PAH [209].

Suppression of the JmjC domain‐containing protein JMJD3, which demethylates H3K27me3, has been shown to decrease PASMC proliferation and reduce inflammation in PAH models. Pharmacological inhibition of JMJD3 with GSK‐J4 significantly decreases the production of inflammatory cytokines such as IL‐6 and enhances apoptosis in PAECs, further highlighting the importance of histone demethylation in PAH pathogenesis [230]. According to Crosswhite et al., TNF‐α reduced histone H3 lysine 4 (H3K4) methylation in PASMCs, which is associated with transcriptional activation [210]. This demethylation may involve the LSD1, potentially contributing to TNF‐α‐induced PASMC proliferation and enhancing the overall proliferative response of the cells [210]. Targeting histone modifiers has emerged as a promising therapeutic strategy in PAH due to their ability to regulate key genes involved in vascular remodeling and inflammation. LSD1, an H3K4/K9 demethylase, is currently under investigation in clinical trials for its potential to treat various diseases. Notably, heterozygous knockout of *LSD1* in mice leads to vascular abnormalities such as hypertension and impaired nitric oxide (NO)‐cGMP signaling, a pathway that is crucial for pulmonary vascular tone regulation and is also targeted by current PAH therapies [231].

Collectively, histone methylation and demethylation are critical regulators of gene expression in the pulmonary vasculature. Understanding the specific roles of these epigenetic modifications in PH may lead to the development of novel therapeutic interventions aimed at reversing the pathological gene expression patterns driving the disease.

#### 3.3.2 Histone Acetylation and Deacetylation

Histone acetylation occurs on lysine (K) residues at the amino ends of core histones, a process regulated by histone acetyltransferases [232]. This modification plays a crucial role in gene expression by loosening the chromatin structure, thereby enhancing accessibility for RNA polymerase and promoting transcription [233]. Acetylated histones also provide binding sites for bromodomain‐containing proteins (BRDs), which help recruit transcriptional machinery and additional chromatin remodeling factors [232, 234]. Conversely, histone deacetylases (HDACs) remove acetyl groups from histone proteins, leading to chromatin compaction and suppression of gene transcription [235]. In humans, 18 HDACs are classified into four groups based on phylogenetic criteria and function: class I (HDAC1, HDAC2, HDAC3, HDAC8), class II (HDAC4, HDAC5, HDAC6, HDAC7, HDAC9, HDAC10), class III (SIRT1 to SIRT7), and class IV (HDAC11) [235]. HDACs can also deacetylate nonhistone proteins, affecting various cellular functions [235].

In the context of PH, the regulation of histone acetylation and deacetylation significantly impacts disease progression (Table 2). According to Su et al. [236], cigarette smoke‐induced microR‐1249 in endothelial extracellular vesicles downregulates HDAC10 in PASMCs, leading to increased acetylation of *NF‐κB*, its nuclear translocation, and upregulation of calcium‐sensing receptor. This promotes resistance to apoptosis and cell proliferation, contributing to PH development [236]. Also, histone acetylation is linked to the expression of eNOS, a critical factor in the pathogenesis of PPHN [17, 237]. Enhanced acetylation of histones H3 and H4 in the promoter region of the *eNOS* gene has been noted in pulmonary vascular endothelial cells affected by PPHN, indicating a potential mechanism through which histone modification influences PH development [17, 237].

Moreover, some studies illustrate the therapeutic potential of HDAC inhibitors by demonstrating that these agents can enhance histone acetylation, leading to improved outcomes in RVH and pulmonary vascular remodeling [238, 239]. In PAH‐PASMCs, increased acetylation of SIRT1 targets, such as histone H1 and *forkhead box protein O1*, promotes cell proliferation. Conversely, SIRT1 activation with SH3 and cysteine‐rich domain 3 reduces acetylation, inhibits PASMC proliferation, and enhances mitochondrial biogenesis by upregulating PPARγ coactivator‐1α targets [240]. Additionally, in patients with IPAH, HDAC, particularly through class I HDAC3, decreases SOD3 expression in PASMC [241]. Selective class I HDAC inhibitors can enhance SOD3 expression in IPAH PASMC, suggesting their potential protective role by counteracting HDAC [241]. Similarly, Class I HDAC isoforms are overexpressed in IPAH, including HDAC1, HDAC2, and HDAC8, and are associated with the proliferation of pulmonary arterial adventitial fibroblasts (PAAFs). This effect is due to the fact that HDAC2 increases *yes‐associated protein 1* (*YAP1*) and *LEF1* gene expression, while HDAC8 inhibits KLF2 signaling [242]. The inhibitors of isoform HDAC such as CAY10398 and PCI34051 can also play a protective role in both hypoxia‐induced PH rat models and IPAH‐PAAFs [242]. HDAC6, whose abundance and activity are maintained by Heat Shock Protein 90 (HSP90), as found by Boucherat et al. [243] is upregulated in human PAH and experimental PH models and inhibits *Ku70* acetylation, enhancing Ku70‐Bax binding and preventing PAH‐PASMCs from stress‐induced mitochondrial apoptosis. At the same time, they found that pharmacological inhibition of HDAC6 reversed the proproliferative, antiapoptotic, and promigratory effects of HDAC6 in PASMC [243].

Valproic acid (VPA) and its products produce similar effects by inhibiting HDACs. For instance, sodium valproate has been shown to effectively block RVH induced by pulmonary artery banding or MCT injections [238]. VPA, also known as an inhibitor of class I HDACs, rescues the proliferation and inflammation caused by elevated expression levels of HDAC1, HDAC2, and HDAC3 in lung tissue of combined MCT and chronic hypoxia rat model [239]. Elevated levels of HDAC1 and HDAC5 have been observed in the pulmonary systems of patients with IPAH and animal models of hypoxia‐induced PH [244]. Inhibition of HDACs using agents like VPA and vorinostat has been shown to mitigate the growth of hypoxia‐induced PAH in rat models and reduce the proliferation of HPASMCs and endothelial cells [244]. These findings highlight the therapeutic promise of targeting HDACs to ameliorate PH and associated right ventricular (RV) remodeling. However, the results of various studies on the effects of HDAC inhibitors have been inconsistent, potentially due to differences in the timing of treatment initiation and the specific animal models used [245].

Together, histone acetylation and deacetylation play critical roles in the pathogenesis of PH. While HDAC inhibitors represent a promising therapeutic strategy for managing PAH, variations in study designs and models necessitate careful consideration of treatment protocols to optimize clinical outcomes. Understanding the stages and characteristics of RV remodeling may further enhance the efficacy of these pharmacological agents in treating PAH.

### 3.4 Noncoding RNA

Nontranslating RNA molecules are commonly referred to as ncRNAs. Only about 2% of the human genome is responsible for coding proteins, while the remaining portion consists of ncRNAs [246]. Based on their roles, ncRNAs are categorized into two groups: functional ncRNAs, such as miRNAs, circular RNAs, and lncRNAs, and nonfunctional ones, sometimes called junk RNAs. Functional ncRNAs play a crucial role in regulating cellular pathways, thus influencing the onset and progression of various diseases. In recent decades, the functions of certain ncRNA types, particularly miRNAs and lncRNAs, have been extensively explored in the context of disease mechanisms, including PAH [17, 20, 247].

#### 3.4.1 MicroRNA

miRNAs are small, ncRNA molecules (∼22 nucleotides) that regulate gene expression at the posttranslational level, playing essential roles in both development and various physiological processes [248]. The miRNA maturation process begins in the nucleus, where long primary miRNAs (pri‐miRNAs) are processed by the enzyme Drosha into precursor miRNAs (pre‐miRNAs). These pre‐miRNAs are then exported to the cytoplasm, where Dicer further processes them into mature miRNA duplexes. One strand called the guide strand, integrates into the RNA‐induced silencing complex (RISC), while the other, known as the passenger strand, is discarded [249]. miRNAs target the 3′ untranslated regions of mRNAs through their seed sequences, regulating gene expression either by mRNA cleavage or by repressing translation, depending on the degree of complementarity [248]. A standardized naming system for miRNAs, typically using the prefix “mir” followed by a unique number, is in place, with exceptions such as let‐7 and lin‐4 retained for historical reasons [250]. Dysregulated miRNA expression has been linked to various diseases, including diabetes, cardiac hypertrophy, cancer, and PH, with clustered miRNAs often sharing similar functional roles and disease relevance [251].

In particular, the role of miRNAs in PH highlights their potential as therapeutic targets for modulating disease progression. Research shows that miRNAs play a crucial role in regulating the expression of genes associated with vascular remodeling, the proliferation of smooth muscle cells, and inflammation, all of which are key factors in the PH [252, 253, 254]. For example, computational analysis indicates that miR‐125a specifically targets the *BMPR2* gene [254], a key player in vascular health and function. Different miRNAs show varied patterns of regulation, including both upregulation and downregulation, which play a role in the pathophysiology of PH (Table 3).

**TABLE 3:** The role of miRNAs in PH.

miRNA	Activity	Cell/tissue	Model	Effect	Reference
miR‐322	Upregulation	Lung	Hypoxia‐PH and MCT‐PAH	Proliferation, migration	[255]
miR‐451		Lung, PAF, PASMC	Hypoxia‐PH and MCT‐PAH	Proliferation, migration	[255]
miR‐210		Lung	Hypoxia‐PH	Increased mitochondrial bioenergetics, mtROS production, vessel remodeling	[256]
miR‐210‐5p		PASMC	MCT‐PAH	Proliferation	[257]
miR‐130/301 family		PAEC, PASMC	Hypoxia‐PH	Proliferation	[252, 258]
miR‐125a		PAEC	Hypoxia‐PH	Proliferation	[254]
miR‐17/92 cluster		PAEC	IL‐6‐induced	Proliferation	[259]
miR‐1249		PAEC, extracellular vesicles	Cigarette smoke‐PH	Proliferation, antiapoptosis of PASMC	[236]
miR‐146‐5p		PAEC	Hypoxia‐induced HPAECs	Proliferation	[260]
miR‐204	Downregulation	PASMC	Hypoxia‐PH and PAH patients	Proliferation, vascular remodeling	[252, 261]
miR‐424		PAEC, PASMC	Hypoxia‐PH and MCT‐PAH	Proliferation, unchecked proliferation	[252, 265]
miR‐371b‐5p		PASMC	MCT‐PAH	Proliferation, antiapoptosis	[264]
miR‐125a‐5p		PASMC	MCT‐PAH	Proliferation, antiapoptosis, glycolysis, vascular remodeling	[262, 263]
miR‐26a‐5p		PASMC	IPAH patients, hypoxia‐PH	Proliferation, migration, autophagy	[266, 267]
miR‐503		PASMC	MCT‐PAH, Su/Hx‐PAH	Unchecked proliferation	[265]

Abbreviations: PASMC, pulmonary artery smooth muscle cell; PAFs, pulmonary arterial fibroblasts; PAEC, pulmonary artery endothelial cell; MCT, monocrotaline; HPAEC, human pulmonary artery endothelial cell; IPAH, idiopathic pulmonary arterial hypertension; PAH, pulmonary arterial hypertension; PH, pulmonary hypertension; mtROS, mitochondrial reactive oxygen species; Su/Hx, Sugen 5416/hypoxia.

In chronic hypoxia and MCT models, upregulation of miR‐322 and miR‐451 has been observed, which is associated with increased cellular proliferation and migration in the pulmonary vasculature [255]. A study demonstrated that miR‐210 upregulation under hypoxia promotes PH by increasing mitochondrial bioenergetics, mitochondrial ROS production, right ventricular wall thickness, and pulmonary vessel remodeling [256]. At the cellular level, the MCT model of PAH shows upregulation of miR‐210‐5p and downregulation of *ATPase sarcoplasmic/endoplasmic retic Ca^2+^ transporting 2* (*ATP2A2*). The elevated levels of miR‐210‐5p result in reduced ATP2A2 levels, which in turn promotes the proliferation of PASMCs. Furthermore, dual luciferase assays provide evidence that *ATP2A2* is a direct target of miR‐210‐5p, establishing a clear relationship between these molecular alterations and the increased proliferation of PASMCs in this disease context [257]. The miR‐130/301 family is significantly upregulated in multiple PH models and human tissues, where it represses miR‐204 and miR‐424, thereby promoting the proliferation of PASMCs. Similarly, both miR‐125a and the miR‐130/301 cluster are upregulated in PH, leading to decreased levels of *BMPR2* and *PPARγ*, which further enhances PAEC proliferation [252, 258]. In PAECs, upregulation of the miR‐17/92 cluster leads to decreased *BMPR2* levels, promoting PAEC proliferation and contributing to PH pathology [259]. Additionally, the upregulation of miR‐146‐5p increases the expression of ubiquitin‐specific peptidase 3 during hypoxic conditions, contributing to enhanced proliferation of PAECs [260]. Moreover, in cigarette smoke‐exposed rats, the elevation of miR‐1249 in endothelial extracellular vesicles downregulates HDAC10 in PASMCs, leading to increased acetylation of *NF‐κB* and thus upregulation of the calcium‐sensing receptor. This promotes cell proliferation and resistance to apoptosis, contributing to the development of PH [236].

In contrast, downregulated miRNAs in PH include miR‐204, which is crucial for maintaining PASMC quiescence. Its downregulation leads to the activation of proproliferative signaling pathways, such as signal transducer and activator of transcription 3 (STAT3), thereby contributing to vascular remodeling [261]. The reduction of miR‐125a‐5p in the MCT‐induced PAH rat model inhibits TGF‐β1/Smad2/3 and IL‐6/STAT3 signaling pathways, thereby enhancing proliferation and suppressing apoptosis of PASMC [262]. Furthermore, the downregulation of miR‐125a‐5p in PAH results in elevated levels of hexokinase 2 (HK‐II) and STAT3, contributing to enhanced PASMC proliferation [263]. In the MCT‐PAH model, reduced levels of miR‐371b‐5p lead to decreased levels of PTEN, which subsequently promotes PASMC proliferation while suppressing apoptosis [264]. Similarly, miR‐424 and miR‐503 are reduced in PH, resulting in unchecked proliferation and survival of endothelial cells [265]. In hypoxia‐induced PASMC, elevation of HIF‐1α induces downregulation of miR‐26a‐5p, which leads to increased 6‐phosphofructo‐2‐kinase/fructose‐2,6‐biphosphatase 3 and unc‐51 like autophagy activating kinase 1/2, resulting in PASMC autophagy, migration and proliferation [266]. Also, the downregulation of miR‐26a‐5p can be induced by elevated lncRNA AC068039.4 in hypoxic PASMCs. This decrease upregulates transient receptor potential cation channel subfamily C member 6 (TRPC6) expression and thus increases the basal intracellular calcium concentration, leading to cell proliferation and migration [267]. These regulatory patterns underscore the complex involvement of miRNAs in the progression of PH, with potential therapeutic implications.

#### 3.4.2 Long ncRNAs

lncRNAs are RNA molecules longer than 200 nucleotides that do not code for proteins but are crucial for regulating gene expression. They act as scaffolds for chromatin‐modifying complexes and nuclear structures, facilitate long‐range chromatin interactions as enhancers, and regulate miRNAs by acting as sponges or decoys, preventing miRNAs from binding to their target messenger RNAs, thus influencing gene silencing and expression [18, 268, 269]. Various lncRNAs play diverse roles in the initiation and progression of PH (Table S1). While some hinder PH development, others actively contribute to its progression, making them promising candidates as biomarkers and therapeutic targets yet to be fully explored. Although evidence has reported the involvement of several lncRNAs, the crucial ones remain unknown. Therefore, in the following sections, we have provided an in‐depth analysis of 27 verified lncRNAs, drawing evidence from literature and databases, and shedding light on their roles, interactions, and potential implications in the pathogenesis of PH. This comprehensive exploration would enrich our understanding of the molecular intricacies underlying PH and pave the way for future therapeutic interventions targeting lncRNAs.

##### 3.4.2.1 lncRNAs Promoting the Development of PH

###### 3.4.2.1.1 Metastasis‐Associated Lung Adenocarcinoma Transcript 1

Metastasis‐associated lung adenocarcinoma transcript 1 (*MALAT1*) located on the human 11q13.1 chromosome was first identified by Ji et al. [270] in 2003 in predicting the metastasis and survival of early non‐small cell lung cancer. The *rs619586A>G* single nucleotide polymorphism (SNP) in *MALAT1* was significantly associated with a decreased PAH risk. It could directly bind to miR‐214 and upregulate X‐box binding protein 1 (XBP1), thereby inhibiting the proliferation and migration of PAECs by shortening the S‐M phase transition [271]. The process of EndMT holds considerable importance in the onset and advancement of PH [272, 273, 274, 275], wherein PAECs undergo a notable transformation, losing polarity and cell‐to‐cell contacts while experiencing extensive cytoskeletal remodeling [274]. MALAT1 plays a crucial role in modulating TGF‐β1‐induced EndMT in human endothelial progenitor cells (EPCs) by regulating *TGFBR2* and *SMAD3* through miR‐145 [276]. Additionally, it modulates ox‐LDL‐induced EndMT in human umbilical vein endothelial cells via the Wnt/β‐catenin signaling pathway [277]. These mechanisms of MALAT1 may contribute to the EndMT of PAECs in PH.

In 2019, Wang et al. [278] found that the expression level of MALAT1 in the pulmonary arteries and HPASMCs of patients with PAH was significantly upregulated. MALAT1 combined with hsa‐miR‐124‐3p.1, inhibited the degradation of target gene *Kruppel‐like factor 5* (*KLF5*), and promoted the proliferation and migration of HPASMCs [278]. KLF5 is a zinc finger transcription factor that acts as the upstream regulator of HIF‐1α, mediating cell survival and migration of HPASMCs through the regulation of various proteins such as cyclin, Bax/Bcl‐2, caspase‐3, and caspase‐9. It plays a key role in hypoxia‐induced vascular remodeling [279]. Undeniably, Courboulin et al. [280] demonstrated that KLF5 is activated in human PAH and implicated in the antiapoptotic and proproliferative phenotype that characterizes PAH‐PASMC.

###### 3.4.2.1.2 H19 Imprinted Maternally Expressed Transcript

H19 imprinted maternally expressed transcript (*H19*) is located on the human 11p15.5 chromosome and is imprinted with the adjacent *insulin‐like growth factor* (*IGF*) *2* gene, a tumor suppressor with abnormal expression in various tumor tissues [281]. Omura et al. [282] found that H19 expression was specifically increased in the RV of both compensated and decompensated PAH patients, as well as in MCT‐induced PAH and pulmonary artery banding rat models. Its expression was positively correlated with cardiac fibrosis and cardiomyocyte hypertrophy. Su et al. [283] found that H19 was highly expressed in the serum and lungs of rats and mice with MCT‐induced PAH and was upregulated in PASMCs after stimulation with the cytokine PDGF‐BB in vitro. *H19* knockout protected mice from MCT‐induced pulmonary artery remodeling and PAH [283]. H19 combined with miRNA let‐7b, upregulated the expression of angiotensin II (AngII) receptor type 1 (AT1R) [283]. AT1R belongs to the G protein‐coupled receptor superfamily. The binding of AngII to AT1R facilitates the coupling of G proteins (Gq/G11 and/or Gi/Go) with its C‐terminal, thereby activating intracellular signaling pathways such as MAPK and RhoA. This activation enhances vascular proliferation and plays a crucial role in PAH [284]. A study reveals that the H19/let‐7g/TGFβR1 axis plays a key role in the development of hypoxic PH by promoting EndMT. H19 deficiency or let‐7g overexpression mitigates pulmonary vascular remodeling and right heart failure, suggesting potential therapeutic targets for hypoxic PH [285]. In PAH, sodium butyrate alleviated RVH by inhibiting H19 overexpression, restoring let‐7g‐5p levels, and suppressing IGF1 receptor/ERK signaling. This resulted in reduced cardiomyocyte hypertrophy and improved cardiac function and survival in MCT‐PAH rats [286]. One study shows that H19 is downregulated in MCT‐induced PAH rats, and melatonin can upregulate it, leading to the binding of H19 with miR‐200a, which subsequently upregulates programmed cell death 4 (PDCD4) and promotes apoptosis of PAMSCs [287]. However, the methodology section of this study lacks sufficient detail, making it difficult to analyze the reasons for discrepancies compared with previous evidence. Based on the available data, there is stronger support for the upregulation of H19 as a factor that promotes PAH. This underscores the need for further research to clarify the underlying reasons for these differences.

###### 3.4.2.1.3 Taurine‐Regulated Gene 1

Taurine‐regulated gene 1 (*TUG1*), located on chromosome 11A1, is a 7.1 kb lnc RNA first found in the retina [288, 289]. TUG1 was significantly upregulated in the pulmonary arteries of PAH patients, hypoxia‐induced PH mice, and PASMCs under hypoxia [290]. Overexpression of TUG1 directly interacted with miR‐328/miR‐374c [290, 291], Foxc1 (forkhead box C1), and the Notch signaling pathway, promoting the proliferation and migration of PASMCs, inhibiting their apoptosis, and accelerating pulmonary vascular remodeling in hypoxia‐induced PH [292]. Moreover, TUG1 has been shown to act as a sponge for miR‐204 [293]. The downregulation of miR‐204 is associated with the severity of PAH and is responsible for the antiapoptotic and proliferative phenotypes observed in PAH‐PASMCs [261, 294]. Lv et al. [295] first compared the differential expression of lncRNA among HPASMCs, HPAECs, and human pericytes (HPCs) under normoxic and hypoxic conditions and found that the expression of TUG1 increased in HPASMCs and HPCs but decreased in HPAECs under hypoxia. Using predictive software, they speculated that TUG1 might interact with miR‐145‐5p to mediate the expression of SRY‐box transcription factor 4 and Bcl2 modifying factor. Additionally, TUG1 may regulate miR‐129‐5p to affect CYP1B1 and valosin‐containing protein or miR‐138‐5p to affect potassium two‐pore domain channel subfamily K member 3 (KCNK3) and ras homolog family member C, thereby contributing to the development of hypoxia‐induced PH [295].

###### 3.4.2.1.4 SRY‐Box Transcription Factor 2 Overlapping Transcript

SRY‐box transcription factor 2 overlapping transcript (*SOX2‐OT*) as an overlapping transcript of SRY‐box transcription factor 2, located on the human 3q26.33 chromosome, was first reported in human and mouse cDNA sequencing by Straussberg et al. in 2002 [296]. Jiang et al. [297] found that SOX2‐OT was highly expressed in the serum of PAH patients and increased in a time‐dependent manner in hypoxia‐induced HPASMCs. SOX2‐OT binds to miR‐455‐3p and upregulates small ubiquitin‐related modifier 1 (SUMO1), promoting antiapoptosis, proliferation, migration, and inflammation in HPASMCs. This contributes to vascular remodeling and PAH [297]. Some studies have shown that SUMO1 can induce autophagy by promoting the assembly of autophagy‐related compounds [298]. In addition, SUMO1 can interact with various transcription factors such as HIF‐1α, regulating cell cycle and vascular remodeling [299].

###### 3.4.2.1.5 MiR222/221 Cluster Host Gene

MiR222/221 cluster host gene (*MIR222HG*), also known as Ang362, located on the human Xp11.3 chromosome, was first identified by Leung et al. [300] in 2013 when evaluating the transcriptome response of vascular smooth muscle cells (VSMCs) to AngII. *Ang362* is the host gene of *miR‐222* and *miR‐221*. It is regulated by Ang and influences cell proliferation while promoting fibrosis in cardiovascular diseases [301]. Wang et al. [302] found that Ang362 was increased in the lung tissue of PAH patients and hypoxic HPASMCs. Ang362, as the transcriptional host, upregulates the expression of miR‐221 and miR‐222, activates the downstream NF‐κB signaling pathway, promotes the proliferation and migration of HPASMCs, inhibits their apoptosis, and triggers an inflammatory reaction, thereby promoting the occurrence and development of PAH [302].

###### 3.4.2.1.6 Hoxa Cluster Antisense RNA 3

Zhu et al. [303] found for the first time that silencing *HOXA‐AS3* in bone marrow mesenchymal stem cells (MSCs) increased bone formation and decreased fat. *HOXA‐AS3* is a highly homologous transcription factor group HOX gene cluster [304]. Zhang et al. [305] measured the upregulation of *HOXA‐AS3* expression in the pulmonary vascular system in hypoxia‐ and MCT‐induced PAH using real‐time qRT‐PCR [305]. The expression of HOXA‐AS3 in the PASMCs after 24 h of hypoxia was upregulated in a time‐dependent manner, almost evenly distributed in the cytoplasm and nucleus [305]. HOXA‐AS3 regulates cell proliferation and cell cycle distribution by upregulating HOXA3 mRNA and protein levels [305]. Li et al. [306] showed that HOXA‐AS3 downregulated the expression of miR‐675‐3p and overexpressed phosphodiesterase 5A (PDE5A) by regulating the miR‐675‐3p/PDE5 axis. This promoted the growth and migration of HPASMCs, inhibited cell apoptosis, and contributed to the development of PAH [306]. PDE5 inhibitors (PDE5Is), including sildenafil, tadalafil, and vardenafil, have been proven to be effective in the clinical treatment of PH [307, 308].

###### 3.4.2.1.7 PAX‐Interacting Protein 1‐Antisense RNA1

Weirick et al. [309] first reported PAX‐interacting protein 1‐antisense RNA1 (*PAXIP1‐AS1*) in 2018, detected in human kidneys and composed of a single exon on chromosome 7q36.2. Jandl et al. [310] found that PAXIP1‐AS1 was upregulated in the pulmonary arteries of patients with IPAH. PAXIP1‐AS1 is enriched in pulmonary arteries without adventitia, with the highest expression observed in parenchymal fibroblasts and PASMCs, followed by adventitial fibroblasts and PAECs [310]. PAXIP1‐AS1 can promote the proliferation and migration of PASMCs and inhibit their apoptosis by regulating the level of downstream target paxillin [310]. As early as 2012, Weith et al. [311] reported that silencing *paxillin* reduced cell adhesion and proliferation in HPASMCs and increased apoptosis. Song et al. [312] found that PAXIP1‐AS1 expression in lung tissue and serum of MCT‐induced PAH rats and hypoxia‐induced HPASMCs was significantly increased, promoting cell viability and migration of HPASMCs through the ETS1 (ETS proto‐oncogene 1, transcription factor)/WIPF1 (WAS/WASL interacting protein family member 1)/RhoA pathway [312].

###### 3.4.2.1.8 AC068039.4

Qin et al. [267] found that AC068039.4 in hypoxia‐induced PASMCs was three times higher than the normal level in PASMCs under both hypoxia and normoxia. They also confirmed the presence of binding sites between AC068039.4 and miR‐26a‐5p, as well as between miR‐26a‐5p and transient receptor potential canonical 6 (TRPC6) [267]. Research shows that the TRPC6 channel participates in the formation of ROC (receiver‐operated Ca^2+^) and SOC (store‐operated Ca^2+^) channels in PASMC and enhances Ca^2+^ signal transduction [313]. AC068039.4 combined with miR‐26a‐5p reduced the degradation of the downstream target TRPC6, leading to an increase in intracellular Ca^2^⁺ levels, which stimulated pulmonary vasoconstriction as well as the proliferation and migration of PASMCs, ultimately contributing to pulmonary vascular remodeling [267].

###### 3.4.2.1.9 Tyrosine Kinase Receptor‐Inducing lncRNA

Tyrosine kinase receptor‐inducing lncRNA (TYKRIL), a previously undiscovered lncRNA, plays a crucial role in maintaining the hyperproliferative phenotype of PASMCs and pericytes, thereby contributing to the pathological inward remodeling of vessels in PAH [314]. Zehendner et al. [314] found that TYKRIL was consistently upregulated in HPASMCs and lung pericytes, both when exposed to hypoxia and when derived from IPAH patients. This upregulation occurred across all conditions induced by pro‐PH factors such as PDGF, IL‐18, and TGF‐β, and regulated by HIF‐1α. RNA‐sequencing analysis on pericytes with *TYKRIL* silencing demonstrated significant downregulation of the tyrosine kinase receptor PDGFRβ [314]. TYKRIL also acted as a decoy, binding strongly with p53 and regulating its activity by disrupting the formation of p53–p300 complexes. This led to an increase in proliferating cell nuclear antigen‐positive cells and a decrease in deoxynucleotide transferase‐mediated dUTP nick end‐label‐positive cells [314]. TYKRIL appears to be a promising therapeutic target for PAH and hypoxia‐induced PH, with its regulation of cell proliferation and apoptosis linked to the modulation of the p53/PDGFRβ axis [314].

###### 3.4.2.1.10 Urothelial Cancer Associated 1

Urothelial cancer associated 1 (*UCA1*), with a length of 2314‐bp, is located on human chromosome 19p13.12 and consists of three exons and two introns [315, 316]. It was first identified in bladder transitional cell carcinoma by Wang et al. in 2006 [317]. UCA1 exhibited the highest expression in hypoxia‐HPASMCs, promoted proliferation, and inhibited their apoptosis through competitive interaction with inhibitor of growth 5 (ING5) for hnRNP I [318]. *MIR495* is the only overlapped gene between the targets of UCA1 and PH. For instance, overexpression of *UCA1* has been shown to inhibit apoptosis of hippocampal neurons by suppressing miR‐495 [319]. Furthermore, a study revealed that inhibiting miR‐495 can improve hemodynamics and vascular remodeling associated with PH [320]. These findings underscore the role of the UCA1‐miR‐495 axis in influencing PH‐associated processes.

###### 3.4.2.1.11 Smooth Muscle Enriched lncRNA

Smooth muscle enriched lncRNA (*SMILR*) on human chromosome 8q24.13 was first reported in the large‐scale identification and characterization of human gene presumptive replacement promoter by Kimura et al. in 2006 [321]. SMILR can combine with CENPF (centromer protein F) mRNA in late mitosis to promote cell proliferation and drive cell cycle progression [322]. Lei et al. [323] observed that *SMILR* expression was elevated in PASMCs from PAH patients and in hypoxia‐induced PH, which also led to the activation of the RhoA/Rho kinase (ROCK) pathway. ROCK is the most typical effector of small G protein RhoA. Abnormal activation of the RhoA/ROCK pathway exists in major cardiovascular diseases such as atherosclerosis, restenosis, hypertension, PH, and cardiac hypertrophy [324]. SMILR directly binds to miR‐141, leading to the overexpression of its target gene, *RHOA*, and contributing to the proliferation and migration of PASMCs induced by hypoxia [323].

###### 3.4.2.1.12 Myocardial Infarction Associated Transcript

LncRNA myocardial infarction associated transcript (*MIAT*) is a transcript related to myocardial infarction, identified by Ishii et al. [325] on chromosome 22q12.1 in 2006 through a large‐scale case‐control association study using haplotype‐based SNP markers. In 2020, Li et al. [326] found that LncRNA MIAT was upregulated and miR‐29a‐5p was downregulated in the HPAECs under hypoxia and in hypoxia‐induced PH rats. In addition, the expression of oxidative stress indicators, such as ROS and malondialdehyde, was significantly increased in hypoxia‐induced PH rats and HPAECs under hypoxia. The proliferation and migration abilities of HPAECs were enhanced, while the key molecules in the nuclear factor erythroid 2‐related factor 2(Nrf2) signaling pathway, such as Nrf2, heme oxygenase 1, and NAD(P)H quinone dehydrogenase 1 were decreased, showing the opposite effect after *MIAT* knockdown [326]. They found that the combination of LncRNA MIAT and miR‐29a‐5p reduced free miR‐29a‐5p and inhibited the Nrf2 pathway, aggravating oxidative stress in hypoxia‐induced PH rats. This led to the proliferation and migration of HPAECs, contributing to hypoxia‐induced PH [326].

###### 3.4.2.1.13 NONRATT015587.2

In 2019, Sun et al. [327] found that NONRATT015587.2 was significantly upregulated in pulmonary arteries during MCT‐induced PAH, as determined by microarray analysis and qRT‐PCR. They reported that NONRATT015587.2 could upregulate the proportion of cells in the S and G2/M phases, promote the proliferation of PASMCs, and contribute to pulmonary vascular remodeling. However, this effect declined after metformin treatment [327]. The same research group further clarified in 2022 that NONRATT015587.2 promotes the expression of PASMC proliferation genes by targeting p21. They found that metformin can upregulate p21 and inhibit PDGF‐BB‐induced PASMC proliferation through NONRATT015587.2 [328].

###### 3.4.2.1.14 MIR210 Host Gene

LncRNA MIR210 host gene (*MIR210HG*), which is elevated in hypoxic PH, plays a significant role in disease progression. A study reveals that increased MIR210HG levels promote the transition from a contractile to a synthetic phenotype in PASMCs under hypoxia by activating the autophagy‐dependent ferroptosis pathway. The knockdown of *MIR210HG* was reversed by HIF‐2α overexpression, with STAT3 identified as a positive regulator of *MIR210HG* transcription under hypoxic conditions. These findings suggest that MIR210HG is a potential target for hypoxic PH intervention and treatment [329].

###### 3.4.2.1.15 Vessel‐Enriched lncRNA Regulated by PDGF‐BB

The lncRNA vessel‐enriched lncRNA regulated by PDGF‐BB (VELRP), identified as a vessel‐enriched lncRNA upregulated by PDGF‐BB, plays a key role in PH by promoting the proliferation of PASMCs. VELRP enhances trimethylation of H3K4 through interaction with WDR5, leading to the upregulation of cyclin‐dependent kinase 1, cyclin‐dependent kinase 2, and cyclin‐dependent kinase 4, driving PASMC hyperproliferation. In vivo studies using rat PH models confirmed VELRP upregulation in the pulmonary arteries, and its knockdown via targeted intervention significantly reduced right ventricular systolic pressure and pulmonary vascular remodeling. These findings highlight VELRP as a potential therapeutic target for PH [330]. In a study, Chen et al. [331] identified the lncRNA VELRP, which is upregulated by PDGF‐BB through c‐Jun N‐terminal kinase (JNK)/p53 signaling and promotes PASMC proliferation. VELRP enhances H3K4 methylation, increasing cyclin‐dependent kinase expression, driving PASMC hyperproliferation, and contributing to the development of PAH [331].

###### 3.4.2.1.16 ENST00000495536

The lncRNA ENST00000495536 (lnc‐536) is upregulated in PASMCs and plays a critical role in mediating hyperproliferation associated with PAH. A study shows that increased levels of lnc‐536 promote a hyperproliferative phenotype by downregulating the antiproliferative homeobox B13 (HOXB13). Knockdown of *lnc‐536* in vivo prevented adverse effects such as increased right ventricular systolic pressure and pulmonary vascular remodeling. Mechanistically, lnc‐536 functions as a decoy for the RNA‐binding protein RBM25 (RNA binding motif protein 25), sequestering splicing factor proline‐and glutamine‐rich and leading to reduced HOXB13 expression, which contributes to smooth muscle hyperplasia in PAH [332]. Similarly, Chinnappan et al. [333] reported that the lncRNA ENST00000495536 is associated with the mRNA HOXB13 in the context of smooth muscle hyperplasia induced by combined exposure to cocaine and HIV transactivator of transcription (HIV‐Tat) in HPASMCs. The study identified this lncRNA–mRNA pair as significant among other differentially expressed pairs, suggesting a potential regulatory role in the proliferation of smooth muscle cells. The analysis implies that ENST00000495536 may influence the expression of HOXB13, contributing to the mechanisms underlying smooth muscle hyperplasia in the development of HIV‐PAH [333].

###### 3.4.2.1.17 Lysine Methyltransferase 2E Antisense RNA 1

The lncRNA lysine methyltransferase 2E antisense RNA 1 (KMT2E‐AS1) is upregulated in PAH and plays a critical role in regulating HIF‐2α activity. KMT2E‐AS1 stabilizes the protein histone lysine N‐methyltransferase 2E (KMT2E), which increases epigenetic modifications, specifically H3K4me3. This process enhances HIF‐2α‐dependent metabolic and pathogenic endothelial activity, creating a positive feedback loop that further promotes HIF‐2α expression. Genetic analysis revealed a single‐nucleotide variant (*rs73184087*) associated with PAH risk, showing an allele‐specific connection to HIF‐2α and long‐range chromatin interactions. Deficiency of *KMT2E‐AS1* protected against PAH in mice, while its overexpression exacerbated PH, highlighting the KMT2E‐AS1/KMT2E axis as a crucial target in PAH management [334, 335].

##### 3.4.2.2 lncRNAs Suppressing the Development of PH

###### 3.4.2.2.1 Growth Arrest Specific 5

Growth arrest specific 5 (*GAS5*) is a nonprotein coding gene, which was first discovered and isolated in 1988 by screening the highly expressed genes in growth arrest cells [336]. Liu et al. [337] first confirmed that GAS5 inhibited the proliferation and migration of VSMCs induced by PDGF‐BB by competing with miR‐21. In hypoxia‐induced PH rats and hypoxic HPASMCs, GAS5 expression was reduced, while miR‐23b‐3p levels increased and KCNK3 expression decreased, which promoted the proliferation and migration of HPASMCs under hypoxic conditions [338]. GAS5 can bind to miR‐382‐3p to inhibit vascular remodeling and autophagy, playing a role in CTEPH [339].

###### 3.4.2.2.2 Cancer Susceptibility Candidate 2

Cancer susceptibility candidate 2 (*CASC2*) is located on human chromosome 10q26.11 and was first reported in human and mouse cDNA sequencing by Straussberg et al. in 2002 [296]. It has been proven to be a tumor suppressor in human malignant tumors [340]. CASC2 was decreased in the pulmonary arteries of hypoxia‐induced PH rats and hypoxic PASMCs. Overexpression of *CASC2* in hypoxic PASMCs inhibited proliferation, migration, and phenotypic transformation, and promoted apoptosis [341]. CASC2 directly binds to miR‐222, inhibiting ING5, and thereby promoting the proliferation and migration of PASMCs [342].

###### 3.4.2.2.3 Ribosomal Protein S4 X‐Linked

In 2020, Liu et al. [343] first reported the identification of a new lncRNA, ribosomal protein S4 X‐linked (*Rps4l*), located on mouse chromosome 6 with a full length of 941 nt. It was significantly downregulated in the pulmonary arteries of hypoxia‐induced PH mice and in hypoxic PASMCs in a time‐dependent manner [343]. Overexpression of *Rps4l* can physically interact with ILF3 (IL enhancer binding factor 3) and enhance its degradation, thereby affecting the accumulation and stability of HIF‐1α mRNA. This process inhibits the proliferation and migration of hypoxia‐induced PASMCs [343]. *Rps4l* is capable of encoding peptides, and its open reading frame sequence encodes a peptide known as RPS4XL (ribosomal protein S4 X isoform‐like). This peptide was purified in vitro based on its amino acid sequence. The study found that RPS4XL interacts with ribosomal protein S6 (RPS6) to inhibit the phosphorylation of RPS6, thereby reducing the proliferation of hypoxia‐induced PASMCs [344]. RPS4XL inhibits the glycosylation of heat shock cognate 71‐kDa protein (HSC70) by binding with HSC70, a key protein that regulates the stress response in PASMCs. This interaction results in the activation of caspase‐1, reducing pyroptosis in PASMCs and slowing pulmonary fibrosis [345].

###### 3.4.2.2.4 Pulmonary Arterial Hypertension Related Factor

LncRNA pulmonary arterial hypertension related factor (*PAHRF*) is located on human 14q13.3 chromosome [346, 347]. Liu et al. [348] discovered that PAHRF was downregulated in the pulmonary arteries of PAH patients and hypoxic HPASMCs. PAHRF binds to miR‐23a‐3p, reducing the availability of free miR‐23a‐3p, which in turn decreases the degradation of its downstream target, mammalian sterile 20‐like kinase 1 (MST1). This process inhibits PASMC proliferation and promotes cell apoptosis [348]. MST1 is a protein encoded by the homologous gene *MST1* of the Drosophila *Hippo* gene in mammals, which regulates organ size and affects cell proliferation, migration, and apoptosis [349]. It has been reported that MST1 plays an important role in inducing apoptosis of VSMCs and mediating vascular remodeling [350]. Kudryashova et al. [351] reported that MST1 interacts with BUB3 (budding uninhibited by benzimidazoles 3) in HPASMCs and adventitial fibroblasts in the pulmonary arteries of PAH. This interaction leads to the aggregation of BUB3 in an ECM and USP10 (mammalian ubiquitin‐specific protease)‐dependent manner. Additionally, the MST1–BUB3 interaction triggers the upregulation of the Akt–mTORC1 pathway (involving the serine‐threonine kinase Akt and rapamycin complex 1), which promotes cell proliferation and survival [351].

###### 3.4.2.2.5 Antisense ncRNA in the INK4 Locus

Antisense ncRNA in the INK4 locus (*ANRIL*), located in the 9p21 region of the human chromosome, is a large antisense ncRNA (403 kb) discovered by Pasmant et al. [352] in the family of melanoma‐neurological system tumor (NST) syndrome. ANRIL is closely related to cancer [353, 354, 355, 356, 357, 358, 359, 360], atherosclerosis [361, 362, 363, 364, 365, 366], and other diseases [367, 368, 369, 370, 371, 372, 373, 374, 375, 376, 377]. Wang et al. [378] showed that the downregulation of ANRIL under hypoxic conditions caused more HPASMCs to transition from the G0/G1 phase to the G2/M+S phase, promoting cell proliferation and increasing HPASMC migration. However, the mechanism remains unknown, and further research is warranted.

###### 3.4.2.2.6 MANTIS

MANTIS (*lncRNA n342419*), an intronic antisense RNA located adjacent to *Annexin A4* (*ANXA4*), was first identified by Leisegang et al. [379]. MANTIS was found to be downregulated in the lungs of patients with end‐stage IPAH and PAECs under hypoxic conditions. Furthermore, PAECs with *MANTIS* knockdown showed impaired ability to form tube‐like structures in culture. Mechanistically, MANTIS directly interacts with BRG1 (Brahma‐related gene‐1), which regulates key endothelial genes, such as SRY‐box transcription factor 18 (SOX18) and SMAD6, that are involved in angiogenesis. Additionally, MANTIS enhances BRG1's ATPase activity by promoting the interaction of BRG1 with its stimulator SWI/SNF‐related BAF chromatin remodeling complex subunit C1 (BAF155), thereby contributing to angiogenesis in MCT‐induced PAH rats [379].

###### 3.4.2.2.7 TCONS_00034812

Bioinformatics analysis and coexpression network construction identified lncRNA‐TCONS_00008552, which showed significantly increased expression in the PAH group, suggesting its involvement in PAH development and its potential as a diagnostic marker [380]. Liu et al. [381] found that the expression of TCON_00034812 in the pulmonary arteries and PASMCs of PAH rats was significantly downregulated, as confirmed by microarray analysis and RT‐PCR. Their research demonstrated that overexpression of *TCONS_00034812* negatively regulated the transcription factor Storkhead box 1, thereby activating the MAPK signaling pathway. This activation inhibited PASMC proliferation and promoted cell apoptosis [381].

###### 3.4.2.2.8 Mathematically Expressed 3

Mathematically expressed 3 (*MEG3*) is located on the human 14q32.3 chromosome and belongs to an imprinted gene of *DLK1–MEG3* imprinted site [382]. Zhu et al. [383] found that MEG3 was downregulated in HPASMCs under hypoxia, promoting their proliferation and migration through the miR‐21/PTEN axis. In another study, increased levels of glucose‐6‐phosphate dehydrogenase led to a decrease in MEG3 expression, which in turn increased the transcription of serum response factor in hypoxic PASMCs, further promoting their proliferation [384]. MEG3 was also significantly downregulated in the lungs of PAH patients, where it triggered PASMC proliferation and migration via the p53 signaling pathway [385]. However, Xing et al. [386] reported a contrasting finding, showing that MEG3 was upregulated in hypoxic PASMCs, where it directly bound to miR‐328‐3p, leading to increased insulin like growth factor 1 receptor expression, promoting PASMC proliferation, and regulating the development of hypoxia‐induced PH. This contradictory phenomenon may be caused by different species of experimental subjects or different experimental conditions.

###### 3.4.2.2.9 LncRNA Regulated by PDGF and TGF‐β

LncRNA regulated by PDGF and TGF‐β (*LnRPT*) was identified as significantly downregulated in rat PASMCs treated with PDGF‐BB and TGF‐β. A similar downregulation of LnRPT was observed in the pulmonary arteries of MCT‐induced PAH rats [387]. Knocking out *LnRPT* in PASMCs was found to be the most effective method for promoting PASMC proliferation. The researchers discovered that PDGF inhibited LnRPT expression through the activation of the phosphoinositide 3‐kinase (PI3K) pathway [387]. Overexpression of *LnRPT*, on the other hand, inhibited the expression of two genes (*Notch3* and *Jag1*) within the Notch signaling pathway, as well as the cell cycle‐regulating gene *Ccna2*, ultimately suppressing the proliferation of PASMCs [387].

###### 3.4.2.2.10 FOXF1 Adjacent Noncoding Developmental Regulatory RNA

The lncRNA *FENDRR* is significantly downregulated in the nucleus of hypoxic PAECs. A study highlights that FENDRR plays a crucial role in inhibiting hypoxia‐induced pyroptosis in HPAECs. The downregulation of FENDRR is associated with the promotion of pyroptosis through its regulatory effects on the *DRP1* gene, particularly by forming an RNA–DNA triplex that increases the methylation of the DRP1 promoter and decreases its transcriptional activity [187]. Zhao et al. [388] reported that the lncRNA *FENDRR* is highly expressed in essential hypertension, while miR‐423‐5p is downregulated, showing a negative correlation between the two. FENDRR influences endothelial dysfunction by promoting cell apoptosis and inhibiting cell proliferation and migration through the miR‐423‐5p/Nox4 axis, with miR‐423‐5p acting as a target of FENDRR and Nox4 as its downstream target [388].

### 3.5 Potential Therapeutic Strategy for lncRNAs in PH

**FIGURE 4:**
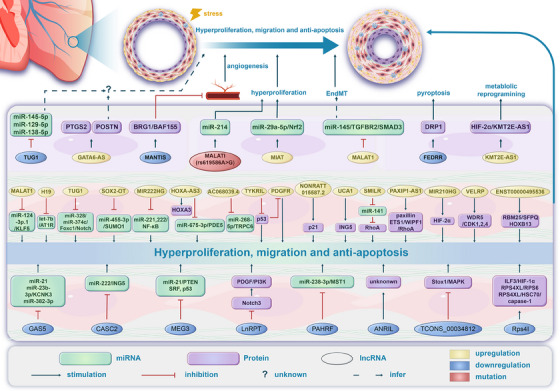
The role of lncRNAs in PH. The literature has established that 27 lncRNAs either promote or inhibit the pathogenesis of PH by interacting with miRNAs or protein molecules. PASMC, pulmonary artery smooth muscle cell; PAEC, pulmonary artery endothelial cell; EndMT, endothelial‐to‐mesenchymal transition; PAH, pulmonary arterial hypertension; PH, pulmonary hypertension.

**FIGURE 5:**
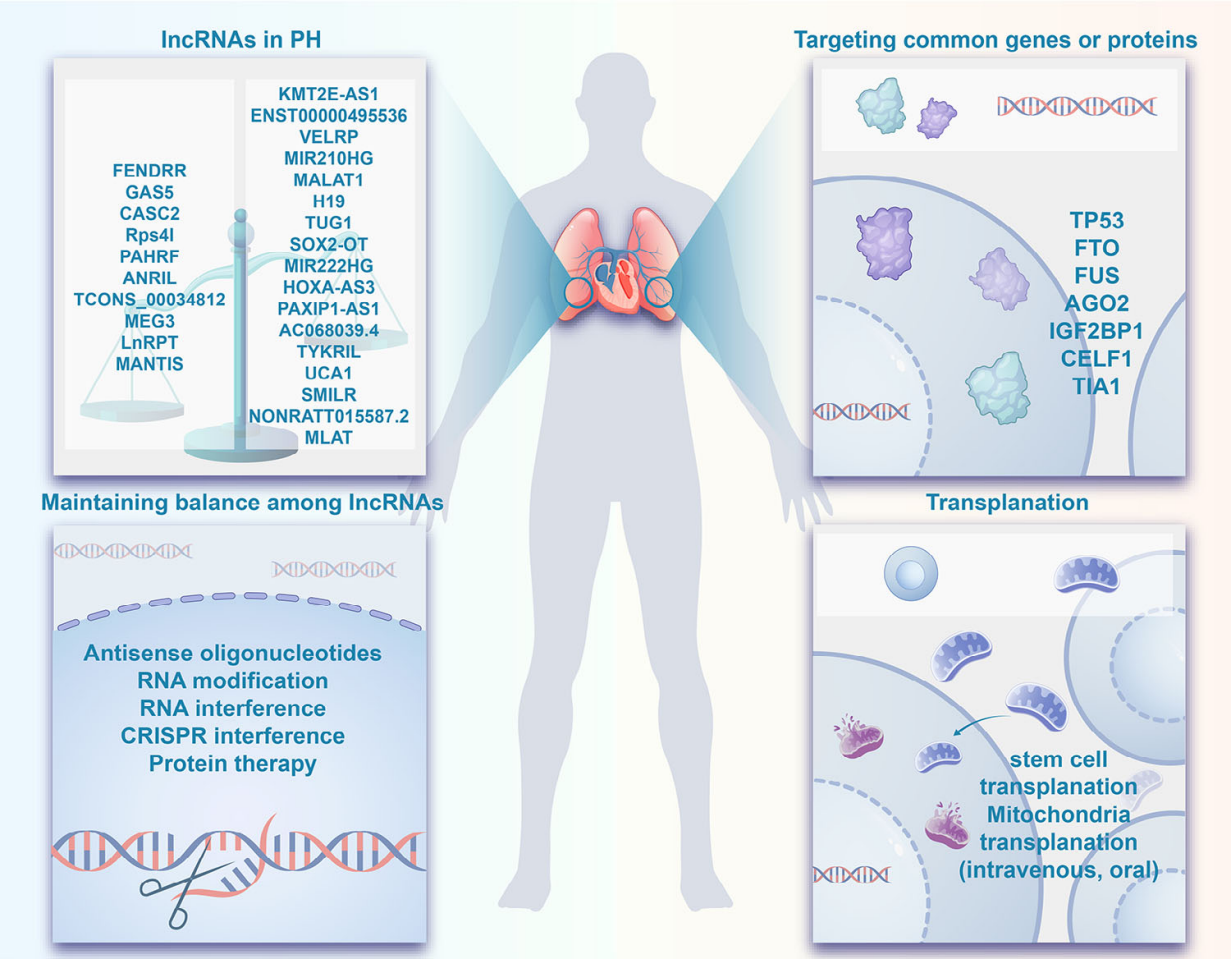
The therapeutic strategies targeting lncRNAs in PH. In PH, specific lncRNAs are upregulated or downregulated, causing lncRNA imbalance and dysregulation of related genes and proteins. Targeting approaches involve regulating associated genes/proteins and balancing lncRNA expression using antisense oligonucleotides, RNA modification/interference, CRISPR, and protein therapy. Emerging transplantation strategies such as stem cell and mitochondrial transplantation (intravenous or oral) are highlighted as potential interventions for restoring cellular homeostasis. PH, pulmonary hypertension; lncRNA, long noncoding RNA; CRISPR, clustered regularly interspaced short palindromic repeats.

This comprehensive review highlights the intricate interplay among vital lncRNAs and their overlapped target genes (Figure S1) during the development and progression of PH. The various lncRNAs played intricate roles in the development and progression of PH, where some are upregulated while others are downregulated (Figure 4 and Table S2), which gives us a clue that these lncRNAs are changed almost simultaneously in PH. Hence, maintaining the balance of these lncRNAs will emerge as a novel potential therapeutic strategy for PH. Additionally, other therapeutic interventions, including targeting overlapped genes influenced by vital lncRNAs, employing stem cells or mitochondrial transplant strategies, or combining two or more of these strategies, may present a promising avenue for future research and clinical applications in the management of PH (Figure 5).

#### 3.5.1 Maintaining Balance Among lncRNAs

Maintaining a delicate balance among key lncRNAs stands out as a crucial therapeutic strategy in addressing PH. This strategic approach involves upregulating certain downregulated lncRNAs and simultaneously downregulating certain upregulated lncRNAs. The equilibrium sought involves a meticulous targeting and modulation of these vital lncRNAs, aiming to restore physiological balance and counteract adverse pathways triggered by the dysregulation of these molecules, which is a hallmark of PH.

The dysregulated expression of lncRNAs in PH contributes to aberrant cellular processes, leading to vascular remodeling and increased PVR. By specifically addressing the dysregulated lncRNAs, therapeutic interventions can potentially rectify these molecular imbalances. Preclinically, the overexpression of key downregulated lncRNAs (GAS5 [339], LnRPT [387], MEG3 [385], CASC2 [341], PAHRF [348]) and the suppression of key upregulated lncRNAs (MALAT1 [278], H19 [282, 283, 389], TUG1 [290, 292], AC068039.4 [267], Ang362 [302], HOXA‐AS3 [306], SMILR [323], LINC00963 [390], and PAXIP1‐AS1 [312]) have been shown to alleviate pathological processes during PH development due the maintenance of physiological balance. Strategies including the use of antisense oligonucleotides (ASOs) [391, 392, 393], RNA modification [394], RNA interference (RNAi) [395], CRISPR interference (CRISPRi) [396], and protein modulations to either inhibit or enhance the expression of specific lncRNAs [397] can aid in the achievement of this physiological balance.

##### 3.5.1.1 Antisense Oligonucleotides

ASOs offer a promising therapeutic strategy for modulating the expression of lncRNAs in PH, as evidenced preclinically in other conditions [391]. ASOs, designed to specifically target dysregulated lncRNAs, exert their influence through mechanisms such as transcriptional modulation, RNA degradation, and splicing modification [398, 399]. In the context of PH, where aberrant lncRNA expression contributes to pathogenesis, ASOs present a precise means of correcting this dysregulation and maintaining a physiological balance. Their potential to be personalized for individual patients and inclusion in combination therapies enhances their appeal. However, challenges such as efficient delivery to target tissues and minimizing off‐target effects necessitate careful consideration.

##### 3.5.1.2 RNA Modification

RNA modification represents an emerging therapeutic target for modulating lncRNA expression in various conditions, including PH [394]. N7‐methylguanosine and other modifications can influence lncRNA stability, localization, and function [394, 400]. By manipulating these modifications, it may be possible to regulate the activity and expressions of specific lncRNAs implicated in PH. This targeted approach holds promise for developing precise interventions to correct dysregulated lncRNA expression and restore cellular balance, offering a unique avenue for therapeutic exploration in the complex regulatory landscape of diseases like PH. However, research in this field is still evolving, and challenges such as specificity and delivery methods need to be addressed for effective and safe therapeutic applications.

##### 3.5.1.3 RNA Interference

RNAi stands as a promising therapeutic avenue for targeting lncRNAs in PH. This approach utilizes small RNA molecules, such as siRNAs or short hairpin RNAs, to selectively silence the expression of specific lncRNAs implicated in PH pathogenesis. By harnessing the cell's natural RNA degradation machinery [401], RNAi can potentially mitigate the dysregulation of key lncRNAs involved in vascular remodeling and PVR, as shown by various preclinical studies [267, 302, 312, 323, 402]. This targeted strategy holds therapeutic potential for correcting the molecular imbalances associated with PH and represents a valuable focus for ongoing research in the field of PH therapeutics.

##### 3.5.1.4 CRISPR Interference

In recent times, the CRISPRi technique for gene silencing has arisen as a compelling substitute for RNAi, demonstrating superior efficiency and precision compared with the latter [403]. CRISPRi holds promise as a therapeutic target for lncRNAs in PH, as shown preclinically in PH [314] and other conditions [404, 405, 406]. In CRISPRi, the CRISPR‐Cas9 system is adapted to act as a programmable transcriptional repressor rather than a genome editor. Specifically, designed guide RNAs are employed to target and bind specific DNA sequences associated with dysregulated lncRNAs [407]. By doing so, CRISPRi can modulate the transcription of these lncRNAs, offering a precise means to influence their expression levels. This targeted approach may hold therapeutic potential in PH by addressing the dysregulation of key lncRNAs implicated in the pathogenesis of the disease, providing a novel avenue for exploring the treatment of PH at the molecular level.

##### 3.5.1.5 Protein Therapy

Protein modulations present a promising therapeutic avenue for regulating lncRNA expression in PH. Li et al. [389] demonstrated that FGF21 administration attenuated hypoxia‐induced PH via the modulation of H19. By targeting proteins involved in transcriptional regulation, chromatin modification, or RNA processing, it becomes possible to influence the expression levels and activity of specific lncRNAs associated with PH pathogenesis [236, 408, 409]. This targeted approach can restore balance in the intricate regulatory networks involved in PH, offering potential interventions to mitigate vascular remodeling, mitochondria dysfunction, and other processes linked to the disease. Protein modulations as a therapeutic strategy hold the promise of addressing the complex molecular landscape of PH and may pave the way for innovative treatment options.

#### 3.5.2 Targeting Overlapped Genes and Their Encoded Proteins

Our analysis of lncRNA subnetworks in PH reveals several overlapped target genes that could serve as potential therapeutic targets by mitigating the adverse effects of the dysregulated lncRNAs.

##### 3.5.2.1 Tumor Protein p53

One prominent target is tumor protein p53 (*TP53*), a vital tumor suppressor gene that regulates cell cycle progression, DNA repair, and apoptosis [410, 411, 412]. *TP53* is often called the “guardian of the genome” because it helps maintain genomic stability [413, 414, 415]. Its identification within the MALAT1/H19/MIAT/MEG3 and MALAT1/H19/MIAT/MEG3/GAS5 subnetworks underscores its significant role in PH pathogenesis. *TP53* is responsible for encoding the p53 protein [416] and its downregulation has been consistently implicated in PH [417, 418, 419, 420, 421, 422, 423, 424, 425], contributing to mitochondrial dysfunction, as observed in numerous studies [411, 426–436]. In preclinical studies, various therapeutic interventions have shown promise in targeting p53 across diverse pathological conditions, including PH. These interventions include glucagon like peptide 1 analogs like exendin‐4 [437], flavonoids (quercetin, curcumin, epigallocatechin gallate) [438, 439, 440], and hormonal regulators such as triiodothyronine [441] and aldosterone [442]. Paracrine factors like paracrine IGF1 [443], modulation of the bradykinin B2 receptor [411, 444, 445], and targeting transcriptional regulators like PPARα [446], PPARγ [417], and nuclear receptor subfamily 4 group A member 1 [410, 447] have shown potential. Peptide‐based interventions, specifically PDRL23a (peptide derived from ribosomal protein L23a)‐derived peptides [448], and small molecules like pifithrin‐α [449, 450, 451, 452, 453, 454, 455] and Nutlin‐3a [456] contribute to the array of strategies. Additionally, Qili Qiangxin capsule [457] represents traditional medicine, and MSCs [458] represent cellular‐based interventions. Collectively, these diverse interventions offer a spectrum of approaches for mitigating p53‐related effects in various pathological conditions, including PH. The potential use of p53 inhibition in cardiovascular diseases raises concerns about its applicability, particularly given its role as a tumor suppressor in other tissues. Long‐term treatments may face limitations, emphasizing the need for caution. Identifying specific stages and times for p53 inhibition is crucial, suggesting its suitability for acute rather than prolonged interventions [410, 459]. While targeting *TP53* or its encoded protein for therapeutic purposes is considered, caution is warranted due to p53's involvement in essential cellular functions.

##### 3.5.2.2 Fat Mass and Obesity‐Associated

Another noteworthy target is fat mass and obesity‐associated (*FTO*), identified within the SOX2‐OT/MIR222HG/HOXA‐AS3/TUG1, and SOX2‐OT/MIR222HG/HOXA‐AS3/TUG1/CASC2 subnetworks. FTO is an RNA demethylase associated with RNA modification processes. Its commonality among these lncRNAs suggests potential relevance in PH. Exploring strategies to modulate FTO activity or expression may offer therapeutic avenues in attenuating the adverse effects of the dysregulated key lncRNAs in this subnetwork. The *FTO* gene encodes a protein that is also named FTO [460]. This protein is a member of the AlkB family, which includes enzymes involved in DNA and RNA demethylation [461]. The FTO protein has been identified as an RNA demethylase, specifically targeting m6A, an overlapped RNA modification [462]. Studies have also shown the implications of FTO in mitochondria dysfunction [463, 464, 465, 466, 467], a hallmark of PH. In the context of PH, heightened FTO expression has been demonstrated to disrupt cyclin D1 stability by modulating the levels of m6A in cyclin D1. This disturbance leads to cell cycle arrest and stimulates cell proliferation, contributing to the initiation and progression of pulmonary vascular remodeling in PH. This implies that targeting the FTO protein could be a promising strategy for intervention in PH, as it plays a crucial role in the dysregulation of cell cycle processes associated with PH [468]. Clinical studies indicate that insulin sensitivity [469] and engagement in physical activity [470, 471, 472, 473, 474, 475, 476] have been observed to lessen the impact of the FTO genotype on obesity‐related characteristics. Furthermore, in preclinical studies, diverse therapeutic interventions exhibiting potential in modulating FTO across various pathological conditions can be categorized based on their nature. Metabolism‐related agents, such as R‐2‐hydroxyglutarate [477] and branched‐chain amino acids [478], have shown promise in influencing FTO function. Additionally, RNA modulators like miR‐192 have been explored for their impact on FTO [479]. Signaling molecules, exemplified by FGF2, have also demonstrated potential in FTO modulation [480]. Chemical compounds, including FB23‐2 [481, 483], meclofenamic acid [482], curcumin [484], and cinnamic acid [463], show promising results in influencing FTO across different contexts. Furthermore, traditional Chinese medicine‐based interventions, such as Qiteng Xiaozhuo granules medicated serum [483] and CangFu Daotan Decoction [485], present additional avenues for exploring FTO modulation in various pathological scenarios. However, careful consideration is essential when targeting the *FTO* gene and its encoded protein to avoid unintended consequences given its involvement in cellular RNA processes.

##### 3.5.2.3 Fused in Sarcoma

Fused in sarcoma (FUS) is an RNA‐binding protein that plays a role in RNA processing and transport [486]. In the context of PH, *FUS* has been identified as an overlapped target gene within interconnected lncRNA subnetworks including the SOX2‐OT/MIR222HG/HOXA‐AS3/TUG1, GAS5/CASC2/MEG3, and SOX2‐OT/MIR222HG/HOXA‐AS3/TUG1/CASC2. Targeting *FUS* as a therapeutic strategy in PH may involve modulating its expression or activity to influence the regulatory network it participates in. The exact mechanisms through which FUS contributes to PH pathology are still an active area of research, but its involvement in RNA‐related processes suggests its potential significance in cellular dysregulation associated with pulmonary vascular remodeling [487, 488, 489]. Preclinically, curcumin [490], therapeutic modulation of glutathione S‐transferase omega 2 [491], overexpression of miR‐141‐3p [492], miR‐4319 [493], and miR‐4478 [494] have shown great promise in targeting *FUS* in various conditions. Developing interventions targeting *FUS* could offer a novel approach to disrupt or mitigate the molecular pathways contributing to PH. Further research is needed to elucidate FUS's precise role in PH and explore the therapeutic potential of targeting this RNA‐binding protein in the context of PH.

##### 3.5.2.4 Other Targets

Targeting the remaining overlapped genes, such as *Argonaute RISC catalytic component 2* (*AGO2*), *IGF2BP1*, *CUGBP Elav‐like family member 1* (*CELF1*), and *TIA1 cytotoxic granule associated RNA binding protein* (*TIA1*), within the described lncRNA subnetworks presents potential therapeutic avenues for addressing PH. *AGO2*, a key player in RNAi, could be targeted to influence posttranscriptional gene regulation [495, 496] and mitochondrial function [497]. *IGF2BP1* is an RNA‐binding protein [498], and strategies for this gene might involve modulating its activity or expression to impact the stability and translation of mRNAs relevant to vascular remodeling. *CELF1*, involved in mRNA splicing [499], could be a target for interventions to influence alternative splicing events in genes crucial for pulmonary vascular function. Additionally, considering *TIA1*'s involvement in stress granule formation and the regulation of mRNA translation [500], it stands as a potential therapeutic target for mitigating the cellular stress response in the context of PH. This is particularly noteworthy as *TIA1*'s modulation could extend therapeutic benefits by influencing the expression of *TP53* [500], a gene commonly shared within the lncRNA subnetworks. Developing targeted interventions for these overlapped genes may contribute to a comprehensive approach for managing PH, addressing diverse aspects of RNA‐related processes implicated in the pathology of this condition. The identification of these overlapped target genes provides valuable insights for developing targeted interventions aimed at mitigating the dysregulation of lncRNAs and associated pathways in PH.

#### 3.5.3 Transplantation

##### 3.5.3.1 Stem Cell Therapy

Stem cells stand out as among the most clinically viable alternative treatments for various diseases [501]. The varied and advancing capabilities of these cells, which include immunomodulatory effects, anti‐inflammatory actions, enhancement of mitochondrial function, and regenerative potential, render them a promising avenue for therapeutic intervention in numerous human diseases [501, 502, 503, 504]. Noteworthy research findings highlight that MSCs, a subtype of adult stem cells, exhibit the capacity to treat various diseases [501, 505, 506], including PH [507, 508, 509]. This implies a compelling prospect for harnessing stem cell therapies to actively shape the expressions of proteins, lncRNAs, and their target genes, thereby unveiling novel therapeutic opportunities for addressing PH.

##### 3.5.3.2 Mitochondrial Transplantation

Considering the pivotal role of mitochondria in the pathogenesis of PH, a proposed therapeutic strategy involves not only maintaining the balance of lncRNAs and modulating overlapped genes and their encoded proteins but also implementing mitochondrial transplant therapy. This innovative approach aims to replace or augment damaged mitochondria with healthy ones, potentially restoring cellular function and enhancing overall vascular performance [411, 510].

In a pioneering investigation, Emani et al. [511] successfully utilized mitochondrial transplantation for patients experiencing myocardial ischemia/reperfusion (I/R) injury. They isolated and purified mitochondria from the skeletal muscle of these patients, subsequently transplanting them into the hearts undergoing ischemia [288]. This intervention led to a significant improvement in cardiac function, marking a noteworthy advancement in addressing myocardial I/R injury through mitochondrial transplantation [511] which has been supported by a pilot study [512]. Subsequent in vitro experiments substantiated the effectiveness of transplanting purified mitochondria, demonstrating a decline in hypoxia‐induced production of ROS in mitochondria and the prevention of hypoxia‐induced pulmonary vasoconstriction in rats [513]. Furthermore, studies utilizing rat models of chronic hypoxia‐induced PAH disclosed that intravenous transplantation of mitochondria lessened PVR [514]. Administering mitochondria orally also exhibited potential in ameliorating PAH in rats exposed to hypoxia and MCT induction [515]. These findings highlight the promising role of mitochondrial transplantation as a therapeutic strategy, particularly in cardiovascular diseases and PAH. However, it is essential to note that mitochondrial transplant therapy is still in its early stages of research [516], and further clinical trials and studies are necessary to establish its safety and efficacy for treating PH.

### 3.6 Translational Value and Challenges of lncRNAs in PH

Unlike protein‐coding genes, lncRNAs exhibit high tissue and cell‐type specificity [517, 518], making them ideal candidates for precision medicine. For instance, in PH, although both lncRNA H19 and MALAT1 exhibit higher pulmonary vascular tissue expression [519, 520], H19 is highly specific to PASMCs, driving their pathological proliferation and resistance to apoptosis [283], while MALAT1 is specific to PAECs, where it regulates endothelial dysfunction, angiogenesis, and inflammation [521, 522, 523]. By targeting these lncRNAs, therapies can modulate disease pathways with greater specificity, offering more personalized treatment options and potentially overcoming the limitations of current symptomatic treatments.

LncRNAs also hold significant promise in advancing the diagnosis, classification, risk stratification, and prognosis of PH. Altered levels of lncRNAs in the blood have been proposed as a potential novel diagnostic biomarker for PH [524, 525]. Unique lncRNA expression profiles in circulation could help categorize PH into different subtypes, offering valuable insights into the underlying disease mechanisms and informing treatment approaches. Additionally, these profiles could aid in risk stratification, particularly when used alongside other established biomarkers. Specifically, circulating lncRNAs such as H19 have been shown to differentiate PAH patients from controls, correlate with RV function, and predict long‐term survival in two independent IPAH cohorts [282]. Moreover, H19 levels, when combined with N‐terminal proBNP (NT‐proBNP) levels or risk scores from the Registry to Evaluate Early and Long‐term PAH Disease Management (REVEAL registry) and the 2015 European Pulmonary Hypertension Guidelines, help identify subgroups of patients with distinct prognoses [282]. For risk stratification, lncRNAs associated with disease severity or progression, such as those involved in hypoxia‐responsive pathways, could help identify high‐risk patients. Additionally, lncRNA signatures may serve as prognostic markers, aiding in the prediction of treatment outcomes and long‐term survival [525].

Despite the aforementioned advantages, the clinical translation of lncRNA‐based approaches is not without obstacles. The cell‐type specificity and context‐dependent expression of lncRNAs, while advantageous for targeted therapy, complicate their identification as universally effective targets. Delivery mechanisms for lncRNA‐modulating agents, such as ASOs or siRNAs, face challenges like off‐target effects, immunogenicity, and inefficient delivery to pulmonary tissues [526, 527, 528]. Moreover, preclinical models often fail to accurately predict drug efficacy in humans [529, 530], limiting the validation of therapeutic and diagnostic applications of lncRNAs in PH.

To overcome these barriers, future research should prioritize developing advanced delivery systems, such as lipid nanoparticles or viral vectors, to ensure precise and efficient targeting of lncRNAs in pulmonary tissues. Standardizing protocols for detecting and quantifying lncRNAs will enhance the reliability of diagnostic tools, facilitating early diagnosis and accurate risk stratification. Emerging technologies like single‐cell transcriptomics and spatial profiling can provide a detailed understanding of the cell‐specific and spatial roles of lncRNAs in PH.

## 4 Clinical Evidence and Therapeutic Strategies

### 4.1 Emerging Therapies and Novel Targets in PH Management

Current therapeutic targets for PH focus on PGI2, NO, ET‐1, and TGF‐β pathways. Approved treatments include endothelin receptor antagonists, PDE5Is, soluble guanylate cyclase (sGC) agonists, and PGI2 analogs and receptor agonists. Additionally, ongoing clinical studies are exploring drugs that target mechanisms involved in PH pathogenesis, such as BMP signaling, tyrosine kinase receptors, estrogen metabolism, ECM remodeling, angiogenesis, epigenetics, serotonin metabolism, and guanylate cyclase signaling.

Several clinical studies have investigated tyrosine kinase receptor inhibition as a therapeutic approach for PAH. Frost et al. [531] reported that imatinib mesylate, a tyrosine kinase inhibitor originally used for chronic myeloid leukemia, improved hemodynamics and right ventricular function in a PAH patient. The IMPRES trial (phase 3) evaluated imatinib in PAH patients receiving two or more therapies, finding a 32‐m improvement in the 6‐minute walk test (6MWT) and a 31.8% decrease in PVR, though adverse effects led to a 27% dropout rate. Additionally, subdural hematomas occurred in patients on anticoagulation [531]. Due to these safety concerns, the PIPAH trial is ongoing to determine a tolerable imatinib dose, with the exclusion of anticoagulated patients [532, 533]. Seralutinib, an inhaled tyrosine kinase inhibitor targeting PDGF receptors, is also under investigation. The Torrey Study (phase 2) assessed its efficacy in PAH patients, with endpoints including changes in PVR and 6MWT distance [532, 534]. The outcome shows that inhaled seralutinib significantly reduced pulmonary PVR compared with placebo in patients with PAH, meeting the primary endpoint. Cough was the most common treatment‐emergent adverse event in both groups [535].

Heterozygous *BMPR2* mutations are the most common genetic cause of PAH, contributing to 53–86% of familial and 14–35% of idiopathic cases [23, 202, 536, 537]. Recent studies highlight *GDF2* mutations (BMP9), affecting 6.7% of IPAH cases, leading to reduced BMP9/BMP10 levels and dysfunction in pulmonary endothelial cells [44, 538] Sotatercept, an activin receptor type IIA fusion protein, restores BMPR2/SMAD1/5/8 signaling and counteracts PAH vascular remodeling [539]. In phase 2 (PULSAR) trials, sotatercept improved PVR, 6MWT distance, and NT‐proBNP in patients [532, 540, 541], with adverse events like thrombocytopenia and telangiectasia. Additionally, in the phase 2a SPECTRA study, sotatercept significantly improved peak oxygen uptake, secondary endpoints including hemodynamics and 6‐minute walk distance, and right ventricular function in patients with PAH after 24 weeks of treatment [542]. In the phase 3 STELLAR trial, sotatercept significantly improved exercise capacity, measured by the 6MWT, in patients with PAH on stable background therapy compared with placebo. The treatment group showed a median increase of 34.4 m versus 1.0 m in the placebo group, with a Hodges‐Lehmann estimate difference of 40.8 m. Sotatercept also improved most secondary endpoints but was associated with increased rates of adverse events such as epistaxis, thrombocytopenia, and elevated hemoglobin [543]. In the STELLAR trial, 25.9% of participants developed antidrug antibodies (ADAs) against sotatercept, with 6.8% also showing neutralizing antibodies. However, ADA presence did not significantly impact the pharmacokinetics, efficacy, or safety of sotatercept in treating PAH [544].

The role of estrogen in PAH has gained significant attention due to its higher incidence in women and the impact of certain estrogen metabolites on cell growth and pulmonary vascular remodeling. Clinical studies have explored the therapeutic potential of antiestrogen drugs traditionally used for breast cancer treatment, such as anastrozole and tamoxifen, in PAH patients. A clinical trial involving 18 male and female PAH patients treated with anastrozole (1 mg/day) for 12 weeks reported significant reductions in estradiol levels and a modest improvement in the 6MWT distance, increasing by 26 m compared with placebo. However, there were no significant changes in right ventricular function or quality of life, and invasive hemodynamics were not assessed [545]. In the phase 2 PHANTOM trial, anastrozole did not significantly improve the 6MWT compared with placebo in postmenopausal women and men with PAH after 6 months. The placebo‐corrected treatment effect was −7.9 m, with no significant differences in adverse events between groups [546]. Tamoxifen, an estrogen receptor blocker, is also being assessed in a phase 2 trial focusing on its effects on PAH patients who are already receiving background therapy. The primary endpoint of this study is the change in tricuspid annular plane systolic excursion, with secondary endpoints including 6MWT distance and quality of life [532, 547]. These trials are critical in understanding the clinical implications of targeting estrogen signaling in PAH, despite the complexities and paradoxes surrounding estrogen's role in this disease.

Elafin, a potent inhibitor of elastases and matrix metalloproteinases, shows promise in preventing pulmonary vascular remodeling by maintaining ECM integrity. A phase 1 clinical trial assessing the safety, tolerability, and pharmacokinetics of elafin in healthy subjects has been completed, with plans for a phase 2 trial in severe PAH currently underway [532, 548].

The deficiency of eNOS contributes to reduced NO production in PAH, leading to endothelial dysfunction and vascular remodeling. Clinical studies have explored the therapeutic potential of EPCs transfected with eNOS (EPC–eNOS) in PAH patients. A phase 1 trial, known as PHACeT, demonstrated the safety of EPC–eNOS administration and significant improvements in the 6MWT at 1, 3, and 6 months, although no sustained hemodynamic benefits were observed [532, 549, 550]. Currently, the ongoing SAPPHIRE trial is investigating the efficacy of EPC–eNOS in PAH, with a focus on exercise capacity and RV function [532, 551]. These studies are essential in understanding the clinical implications of targeting eNOS and EPCs in the management of PAH.

Research into the role of epigenetics in PAH has highlighted BRD4 as a significant factor regulating gene expression associated with PAH pathogenesis [221, 552]. Based on promising preclinical findings, the 16‐week pilot study APPRoAcH‐p, initiated in 2018, evaluated the safety and hemodynamic effects of oral apabetalone, a bromodomain and extraterminal inhibitor, in PAH patients classified as functional classes 2 or 3 receiving standard therapy. The study has been completed and aimed to assess whether apabetalone could effectively improve hemodynamics and overall patient outcomes in this population [532, 553].

Bardoxolone methyl is a potential therapy for PAH that reduces oxidative stress and NF‐κB activation by activating Nrf2, promoting antioxidant gene transcription, and enhancing oxidative phosphorylation [554]. The LARIAT phase 2 clinical trial showed interim improvement in patients with connective tissue disease‐associated PAH (CTD‐PAH) [555, 556]. This prompted two follow‐up trials, CATALYST and RANGER, which were later terminated due to COVID‐19 concerns [557, 558]. Further research is needed to explore bardoxolone's therapeutic role in CTD‐PAH and other forms of PH.

Rodatristat ethyl is an orally bioavailable, reversible inhibitor of TPH, aimed at reducing peripheral serotonin production without affecting the central nervous system. Patients with PAH exhibit elevated free serotonin levels, contributing to pulmonary vasoconstriction and vascular remodeling [559]. Rodatristat ethyl has been tested in the completed ELEVATE2 clinical trial, a placebo‐controlled, randomized study evaluating doses of 300 and 600 mg twice daily for 24 weeks [560]. The study found that Rodatristat ethyl (300 and 600 mg) significantly reduced PVR at week 24, with mean changes of 63.1% and 64.2%, respectively, compared with 5.8% for placebo. Adverse events occurred in 92% of the 300 mg group and 100% of the 600 mg group, with discontinuation rates of 11% for both RE doses. One death was reported in the 300 mg group [561].

In the phase 1 clinical trial, MK‐5475, a selective inhaled sGC stimulator, was evaluated for safety and pharmacokinetics/pharmacodynamics in participants with PH associated with COPD (PH‐COPD). The trial included 23 participants, with a median age of 68.5 years and 86.4% being male, who were randomized to receive either MK‐5475 or a placebo. The results demonstrated a favorable safety profile, with similar rates of adverse events in both groups. While MK‐5475 did not significantly affect arterial blood oxygenation or pulmonary blood volume, it resulted in a numerical reduction in PVR of −21.2% compared with −5.4% in the placebo group. These findings suggest potential efficacy for MK‐5475 and support its further development as a treatment for PH‐COPD [562].

### 4.2 Efficacy and Safety of Approved Pharmacological Therapies in PH

The KABUKI trial provided important insights into the efficacy and safety of edoxaban compared with warfarin in patients with CTEPH following reperfusion therapy. The study found that edoxaban was noninferior to warfarin in preventing worsening PVR over 48 weeks, with no incidents of symptomatic venous thromboembolism. Clinically relevant bleeding events occurred at similar rates in both groups, suggesting that edoxaban may serve as a viable alternative for anticoagulation in this patient population [563].

In the MERIT‐1 trial, macitentan demonstrated a significant reduction in PVR, achieving 71.5% of baseline compared with 87.6% in the placebo group at week 16 among patients with CTEPH. The most common adverse events associated with macitentan included peripheral edema and decreased hemoglobin [564]. In contrast, the PASSION trial evaluated the efficacy of tadalafil in patients with heart failure with preserved ejection fraction and combined PH. This trial found no benefit in achieving the primary composite endpoint of heart failure hospitalization or all‐cause mortality. Notably, the tadalafil group exhibited a significantly higher all‐cause mortality rate. Due to medication supply issues, the trial was terminated early, and no significant improvements were observed in secondary endpoints [565]. Building on the efficacy of individual therapies, the A DUE study revealed that a fixed‐dose combination of macitentan and tadalafil (M/T FDC) effectively reduced PVR by 29% compared with macitentan alone and by 28% compared with tadalafil alone. Although the M/T FDC group experienced three unrelated deaths and a higher rate of adverse events leading to treatment discontinuation, the findings support the combination as an effective option for simplifying treatment in PAH patients [566].

## 5 Conclusion and Future Perspectives

Despite significant progress in developing new drug targets and the availability of current vasodilator therapies, PH remains a disease with high mortality rates. Recent large‐scale whole exome and whole genome sequencing in extensive patient cohorts have profoundly reshaped our understanding of the genetic underpinnings of Group 1 PAH. Although significant progress has been made, considerable challenges remain. Some of the newly identified genes are linked to established pathways of vascular remodeling and PH, while the functions of others are still not well understood. Epigenetic modifications, including DNA methylation, histone PTMs like acetylation and methylation, and ncRNAs (like miRNA and lncRNA), are involved in multiple aspects of PH, such as abnormal proliferation, apoptosis resistance, regulation of inflammation, and fibrosis, vascular and RV remodeling, and mitochondrial dysfunction. Although certain epigenetic mechanisms require further elucidation, substantial evidence indicates that epigenetic modulators could be promising new therapeutic targets for PH. Extensive research has demonstrated that lncRNA dysregulation plays a significant role in the pathogenesis of PH.

In this review, we have summarized the mechanisms by which 27 different lncRNAs reported to date, are involved in the occurrence and development of PH. Among them, 17 lncRNAs are upregulated and 10 are downregulated during the pathological process of PH. These lncRNAs have the potential to serve as phenotypic biomarkers for the diagnosis and prognosis of PH.

Database analysis shows that MALAT1 regulates 3225 out of 6896 PH‐related downstream target genes (approximately 50%), suggesting its crucial role in the pathogenesis of PH. Targeting MALAT1 could thus represent an effective therapeutic strategy. According to Zhuo et al. [271], functional polymorphisms in MALAT1 increase susceptibility to PAH in the Chinese population. Moreover, a clinical trial provided relevant data on circulating lncRNA levels, highlighting their potential importance as biomarkers for PAH patients [567]. LncRNAs contribute to the occurrence and progression of PH by modulating miRNAs or directly influencing the expression of target genes. Targeting lncRNAs directly presents a promising approach for novel therapeutic interventions.

With advancements in gene therapy technology, upregulated lncRNAs can be inhibited, while downregulated lncRNAs can be restored through overexpression, offering a new strategy for PH treatment. Focusing on the upregulation, downregulation, or overlapped targets of multiple lncRNAs offers a promising approach to intervene in key pathological mechanisms, providing an effective treatment strategy for PH by significantly mitigating its progression.

## Author Contributions

R. T. and Q. H.: conceptualization, writing‐revising and editing, analysis, supervision, project administration, funding acquisition. Y. W. and J. Y.: writing—revising and editing, supervision. R. T., J. A., Q. Y., and X. L.: writing—original draft, visualization. J. J., D. Y., and K. L.: writing and editing—original draft. All authors contributed to the article and approved the submitted version.

## Ethics Statement

The authors have nothing to report.

## Conflicts of Interest

The authors declare no conflicts of interest.

## Supporting information



Supporting Information

## Data Availability

The authors confirmed that all data required to evaluate the conclusions in the paper are present in the article/Supporting Information; further inquiries can be directed to the corresponding author.
