# Supplementary material for: Pulmonary Hypertension: Molecular Mechanisms and Clinical Studies
Source: MedComm (2020). 2025 Mar 10;6(3):e70134. doi: 10.1002/mco2.70134 (PMC11892029; doi:10.1002/mco2.70134)
Supplement: Supplementary file 1 — Supporting Information [file MCO2-6-e70134-s001.docx]

**Pulmonary Hypertension: Molecular Mechanisms and Clinical Studies**

Joseph Adu-Amankwaah^1#^, Qiang You^2#^, Xiaoer Liu^3#^, Jiayi Jiang^3^, Dongqi Yang^4^, Kuntao Liu^4^, Jinxiang Yuan^5*^, Yanfang Wang^6*^, Qinghua Hu^7,8*^, Rubin Tan^1*^

^1^Department of Physiology, Basic medical school, Xuzhou Medical University, Xuzhou, China, ^2^School of Pharmacy, Shandong University of Traditional Chinese Medicine, Jinan, Shandong, China, ^3^ The first clinical medicine college, Xuzhou Medical University, Xuzhou, China,

^4^College of Life Science, Xuzhou Medical University, Xuzhou, China, ^5^Lin He's Academician Workstation of New Medicine and Clinical Translation, Jining Medical University, Jining, China, ^6^State Key Laboratory of Animal Biotech Breeding, Institute of Animal Science, Chinese Academy of Agricultural Sciences, Beijing, China, ^7^Department of Pathophysiology, School of Basic Medicine, Tongji Medical College, Huazhong University of Science and Technology, Wuhan, China, ^8^Key Laboratory of Pulmonary Diseases of Ministry of Health, Tongji Medical College, Huazhong University of Science and Technology, Wuhan, China.

**# Contribute equally**

***Correspondence:**

Rubin Tan (tanrubin11@126.com)

Qinghua Hu (qinghuaa@mails.tjmu.edu.cn)

Yanfang Wang (wangyanfang@caas.cn)

Jinxiang Yuan (yuanjinxiang18@163.com)

**Supplementary materials**

**Table S1. The clinical relevance of LncRNAs in PH patients.**

| **Number** | **LncRNA** | **Resources** | **Expression** | **References** |
| --- | --- | --- | --- | --- |
| 1 | MALAT1 | HPAECs of PAH patients | rs619586A>G | ^271^ |
|  |  | PAs and HPASMCs of PAH patients | ↑ | ^278^ |
| 2 | TUG1 | HPASMCs of PAH patients, hypoxic HPASMCs and HPCs | ↑ | ^290,295^ |
|  |  | hypoxic HPMECs | ↓ | ^295^ |
| 3 | MIAT | hypoxic HPAECs | ↑ | ^326^ |
| 4 | H19 | plasma and right ventricle of PAH patients | ↑ | ^282^ |
| 5 | MIR222HG | lung tissue of patients with PAH and hypoxic HPASMCs | ↑ | ^302^ |
| 6 | PAXIP1-AS1 | PAs of IPAH patients and HPASMCs | ↑ | ^310^ |
| 7 | MEG3 | hypoxic HPASMCs and lungs of PAH patients | ↓ | ^383,385^ |
| 8 | HOXA-AS3 | hypoxic HPASMCs | ↑ | ^306^ |
| 9 | SOX2-OT | the serum of patients with PAH and hypoxic HPASMCs | ↑ | ^297^ |
| 10 | CASC2 | hypoxic HPASMCs | ↓ | ^342^ |
| 11 | UCA1 | hypoxic HPASMCs | ↑ | ^318^ |
| 12 | SMILR | PASMCs of PAH patients and the serum of patients with PAH | ↑ | ^323^ |
| 13 | AC068039.4 | hypoxic HPASMCs | ↑ | ^267^ |
| 14 | PAHRF | PAs of PAH patients and hypoxic HPASMCs | ↓ | ^348^ |
| 15 | GAS5 | hypoxic HPASMCs | ↓ | ^338^ |
| 16 | ANRIL | hypoxic HPASMC | ↓ | ^378^ |
| 17 | TYKRIL | PASMCs and lung pericytes derived from IPAH patients | ↑ | ^314^ |
| 18 | MANTIS | lungs from patients with end-stage IPAH | ↓ | ^379^ |
| 19 | FENDRR | hypoxic HPAECs | ↓ | ^187^ |
| 20 | ENST00000495536 | combined exposure to cocaine and HIV-Tat (C + T) induced smooth muscle hyperplasia in HPASMCs | ↑ | ^333^ |

HPAECs human pulmonary artery endothelial cells, PAs pulmonary arteries, HPASMCs human pulmonary artery smooth muscle cells, PAH pulmonary arterial hypertension, HPCs human pericytes, HIV human immunodeficiency virus, HPMECs human pulmonary microvascular endothelial cells.

**Table S2. The role of 22 LncRNAs in PH.**

| **cell type** | **Expression** | **LncRNA** | **Target Spot** | **Effect** | **References** |
| --- | --- | --- | --- | --- | --- |
| PASMCs | upregulated | MALAT1 | miR-124-3p.1/KLF5 | promotes proliferation and migration | ^278^ |
|  |  | H19 | miRNA let-7b/AT1R | promotes proliferation | ^282,283^ |
|  |  | TUG1 | miR-328, miR-374c/Foxc1/Notch | promotes proliferation and anti-apoptosis | ^290,292,295^ |
|  |  | SOX2-OT | miR-455-3p/SUMO1 | promotes proliferation, migration, anti-apoptosis, and inflammation | ^297^ |
|  |  | MIR222HG | miR-221 and miR-222/NF-κB | promotes proliferation, migration, anti-apoptosis, and inflammation | ^302^ |
|  |  | HOXA-AS3 | HOXA3, miR-675-3p/PDE5 | promotes proliferation, migration, and anti-apoptosis | ^305,306^ |
|  |  | PAXIP1-AS1 | paxillin、ETS1/WIPF1/RhoA | promotes proliferation, migration, and anti-apoptosis | ^310,312^ |
|  |  | AC068039.4 | miR-26a-5p/TRPC6 | promotes proliferation, migration, and pulmonary vasoconstriction | ^267^ |
|  |  | TYKRIL | p53/PDGFRβ axis | promotes proliferation and anti-apoptosis | ^314^ |
|  |  | UCA1 | ING5 | promotes proliferation and anti-apoptosis | ^318^ |
|  |  | SMILR | miR-141/RhoA | promotes proliferation and migration | ^323^ |
|  |  | NONRATT015587.2 | p21 | promotes proliferation | ^327,328^ |
|  |  | MIR210HG | HIF-2α | promotes autophagy-dependent ferroptosis and synthetic phenotypic transformation | ^329^ |
|  |  | VELRP | WDR5/CDK1, CDK2 and CDK4 | promotes proliferation | ^330^ |
|  |  | ENST00000495536 | RBM25/SFPQ/HOXB13 | promotes proliferation and induces smooth muscle hyperplasia | ^332,333^ |
|  | downregulated | GAS5 | miR-21, miR-23b-3p/KCNK3, miR-382-3p | promotes proliferation, migration, autophagy, and vascular remodeling | ^337-339^ |
|  |  | CASC2 | miR-222/ING5 | promotes proliferation, migration, anti-apoptosis, and phenotypic transformation | ^341,342^ |
|  |  | Rps4l | ILF3/HIF-1a, RPS4XL/RPS6, RPS4XL/HSC70/caspase-1 | promotes proliferation, migration, and pyroptosis | ^343-345^ |
|  |  | PAHRF | miR-23a-3p/MST1 | promotes proliferation and anti-apoptosis | ^348^ |
|  |  | ANRIL | Unknown | promotes proliferation and migration | ^378^ |
|  |  | TCONS_00034812 | Stox1/MAPK signaling pathway | promotes proliferation and anti-apoptosis | ^381^ |
|  |  | MEG3 | miR-21/PTEN, SRF, p53 signaling pathway | promotes proliferation and migration | ^383-385^ |
|  |  | LnRPT | PDGF-PI3K-LnRPT-Notch3 | promotes proliferation | ^387^ |
| PAECs | mutation (rs619586A>G) | MALAT1 | miR-214 | inhibits the proliferation and migration, decreases PAH risk | ^271^ |
|  | downregulated | TUG1 | miR-145-5p、miR-129-5p、miR-138-5p | Unknown | ^295^ |
|  |  | MANTIS | BRG1/BAF155 | inhibits angiogenesis | ^379^ |
|  |  | FENDRR | DRP1 | promotes hypoxia-induced pyroptosis | ^187^ |
|  | upregulated | MIAT | miR-29a-5p/Nrf2 pathway | promotes proliferation, migration, and oxidative stress | ^326^ |
|  |  | KMT2E-AS1 | HIF-2α/KMT2E-AS1/KMT2E feed-forward loop | promotes proliferation and orchestrates metabolic reprogramming | ^334,335^ |

PASMCs pulmonary artery smooth muscle cells, PAECs pulmonary artery endothelial cells, PDE5 phosphodiesterase 5, PAH pulmonary arterial hypertension.

**
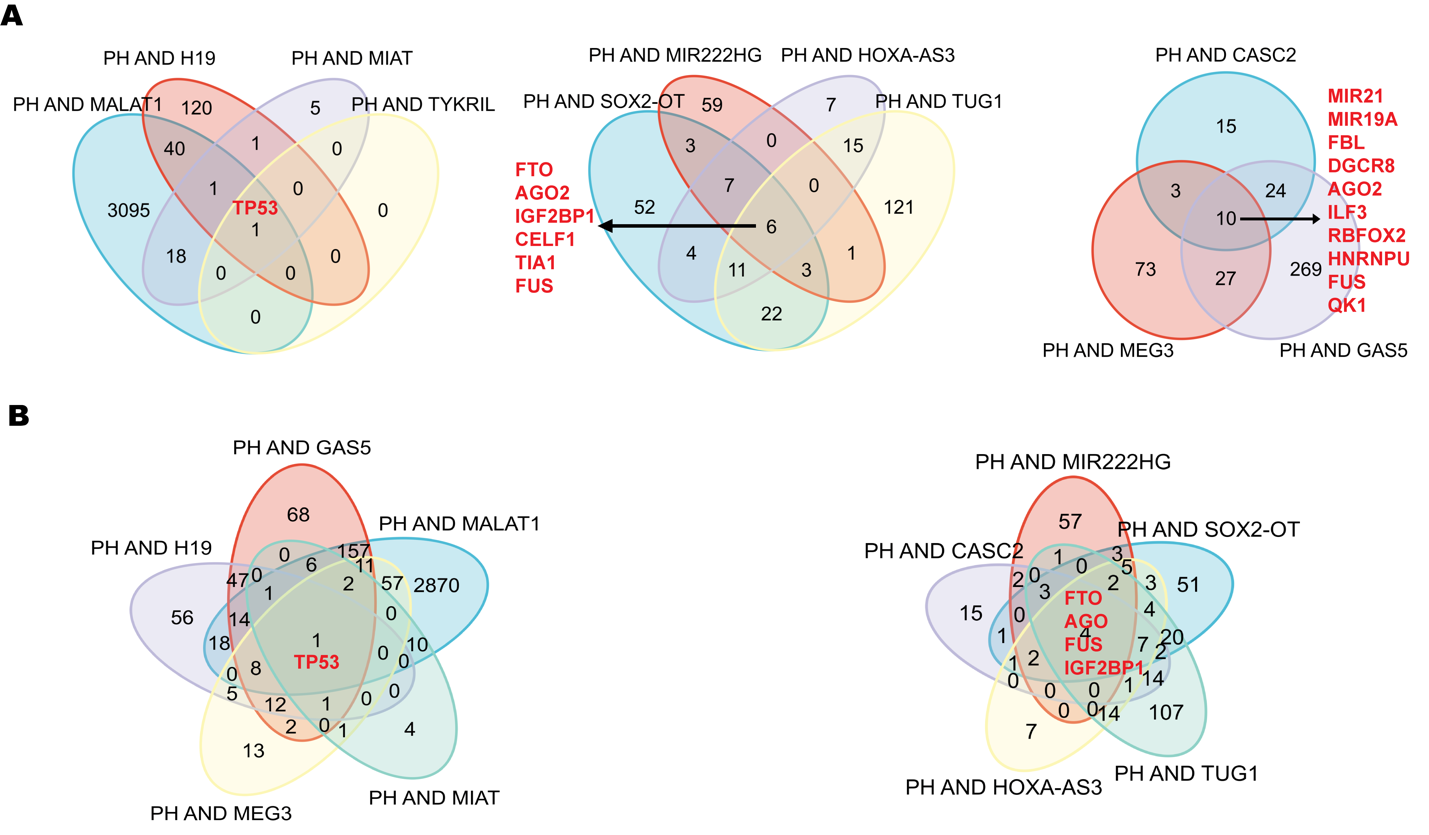
**

**Figure S1. The networks among lncRNAs in PH. A.** The network of MALAT1/H19/MIAT/TYKRIL (*left*), SOX2-OT/MIR222HG/HOXA-AS3/TUG1 (*middle*) and GAS5/CASC2/MEG3 (*right*). **B. The network of** MALAT1/H19/MIAT/MEG3/GAS5 (*left*) and SOX2-OT/MIR222HG/HOXA-AS3/TUG1/CASC2 (*right*). The target genes of LncRNAs were downloaded from the NPInter (http://bigdata.ibp.ac.cn/npinter4/), and ENCORI (https://starbase.sysu.edu.cn) databases. Potential therapeutic target genes for PH were obtained using the GeneCards database (https://www.genecards.org). The Venn diagram was performed using R language 4.0.1(version 3.6.3). PH pulmonary hypertension.
